# Traditional Chinese botanical medicines and plant-derived bioactive compounds in amyotrophic lateral sclerosis: mechanistic evidence, translational challenges, and therapeutic opportunities

**DOI:** 10.3389/fphar.2026.1918967

**Published:** 2026-09-24

**Authors:** Mengfan Li, Ronghao Li, Zhe Deng, Liqun Zhang, Shimeng Lv, Jinjing Shi, Haoteng Ma, Wenhui Guan, Han Zhang, Jiamin Li, Jing Teng

**Affiliations:** 1 The First Clinical Medical College, Shandong University of Traditional Chinese Medicine, Jinan, Shandong, China; 2 Department of Surgery-Transplant Surgery, Johns Hopkins University School of Medicine, Baltimore, MD, United States; 3 Oncology Center-Breast and GYN Malignancies, Johns Hopkins University School of Medicine, Baltimore, MD, United States; 4 Shandong University of Traditional Chinese Medicine Affiliated Hospital, Jinan, Shandong, China; 5 Department of Psychiatry and Behavioral Sciences, Johns Hopkins University School of Medicine, Baltimore, MD, United States

**Keywords:** amyotrophic lateral sclerosis, molecular mechanisms, neuroprotection, phytochemicals, traditional Chinese botanical medicine, traditional Chinese medicine, translational pharmacology

## Abstract

**Introduction:**

Amyotrophic lateral sclerosis (ALS) is a fatal motor neuron disease with limited therapeutic options, highlighting the need for multi-target interventions. Traditional Chinese botanical medicine (TCBM) interventions have attracted increasing attention as potential multi-target pharmacological candidates for ALS. In addition, a separate body of evidence derived from selected plant-derived bioactive compounds directly investigated in ALS-specific models provides complementary mechanistic insights into ALS-related pathways. However, the pharmacological mechanisms and translational potential of these two distinct evidence categories remain insufficiently summarized. This review systematically summarizes ALS-related pathological mechanisms and the molecular actions reported within these two distinct evidence categories, together with translational challenges and future research directions.

**Methods:**

A structured literature search was conducted in PubMed, Web of Science, and ScienceDirect up to May 2026. A total of 198 publications were retained, including 48 ALS-specific original intervention studies forming the core pharmacological evidence set.

**Results:**

ALS involves interconnected pathological processes, including neuroinflammation, oxidative stress, mitochondrial dysfunction, impaired proteostasis, excitotoxicity, and neuromuscular junction injury. Evidence across these two categories suggests modulation of multiple ALS-related pathological processes. Reported effects include regulation of inflammatory signaling, enhancement of Nrf2-mediated antioxidant responses, improvement of PGC-1α/MFN2-associated mitochondrial homeostasis, and promotion of protein clearance pathways.

**Conclusion:**

Together, these distinct but complementary evidence categories highlight the potential for multi-target modulation of ALS-related pathology. However, current evidence remains predominantly preclinical, and clinical efficacy and translational feasibility require further validation.

## 1 Introduction

Amyotrophic lateral sclerosis (ALS) is a rare progressive neurodegenerative disease that primarily affects middle-aged and elderly individuals. It predominantly impacts the motor neurons (MNs) in the cerebral cortex, brainstem, and spinal cord, resulting in gradual muscular weakening and atrophy, speech impairments, and dysphagia. Ultimately, patients frequently succumb to respiratory failure (Feldman et al., 2022). Currently, no curative treatment is available for ALS, and patients often survive for only 3–5 years.

A recent global systematic review and meta-analysis estimated the pooled incidence, prevalence, and mortality of ALS at 1.65 per 100,000 person-years, 5.05 per 100,000 population, and 1.26 per 100,000 person-years, respectively, highlighting the continuing worldwide burden of the disease (Liu R.-Y. et al., 2026). A previous review reported that the overall standardized male-to-female ratio for ALS is 1.28, with its incidence strongly connected to age (Fontana et al., 2021). Due to its high mortality rate and lack of effective treatment methods, ALS has often been described in some reports as a major unresolved therapeutic challenge with substantial public health implications (Zeng et al., 2025).

The etiology and pathogenesis of ALS remain incompletely elucidated. Approximately 5%–10% of ALS cases show an identifiable familial pattern and are conventionally classified as familial ALS (fALS), whereas most cases are apparently sporadic ALS (sALS). Pathogenic or likely pathogenic variants in ALS-associated genes can nevertheless also be identified in a subset of patients without an evident family history (Yuan et al., 2025). It is generally believed that ALS is not caused by a single independent factor, but involves multiple pathophysiological processes such as glutamate excitotoxicity, mitochondrial dysfunction, oxidative stress, neuroinflammation, energy metabolism abnormalities, cytoskeletal disorders, and protein homeostasis abnormalities, and is influenced by environmental factors and genetic background (Schram et al., 2020; Raoufinia et al., 2025).

To date, three major drugs have been approved by the U.S. Food and Drug Administration (FDA) for the treatment of ALS, namely, riluzole, edaravone, and tofersen. Riluzole, an anti-glutamatergic agent, was approved by the FDA for clinical use in 1995. Its pharmacological actions include promoting the inactivation of voltage-gated sodium channels at glutamatergic nerve terminals, activating G protein-dependent signaling cascades, inhibiting postsynaptic glutamatergic transmission, and enhancing postsynaptic γ-aminobutyric acid type A receptor function. These effects may contribute to an extension of survival in patients with ALS by approximately 6–19 months (Corcia et al., 2025). Riluzole is associated with several adverse effects, including nausea, weakness, and transient elevations in liver enzymes (Jaiswal, 2019). Edaravone is an antioxidant and free radical scavenger with antioxidative, neuroprotective, and anti-inflammatory properties. Nevertheless, its therapeutic efficacy in ALS remains limited and may be more evident in selected patient populations, particularly those at an early stage of disease onset with rapid progression. In addition, interethnic differences in treatment response have been reported (Jayasinghe et al., 2022). Tofersen represents the first approved therapy directed against a genetic cause of ALS. As an antisense oligonucleotide, it selectively binds to superoxide dismutase 1 (*SOD1*) messenger RNA (mRNA) and promotes RNase H1-mediated mRNA degradation, thereby reducing the synthesis of mutant SOD1 protein (Jin and Zhong, 2023). Nevertheless, its clinical applicability is restricted to the small subset of patients with *SOD1*-mutant ALS, accounting for approximately 2% of ALS cases. In addition, serious adverse events, including autoimmune myeloradiculitis, have been reported (Wiesenfarth et al., 2024). Collectively, these findings highlight the limitations of current therapeutic options and emphasize the urgent need to develop multi-target and low-toxicity treatment strategies.

Because progressive weakness and muscle atrophy overlap with the manifestations traditionally described as “flaccidity syndrome” (Wei syndrome), ALS is frequently discussed within this traditional Chinese medicine (TCM) conceptual framework (Liu et al., 2023). Within the context of ALS pharmacological research, traditional Chinese botanical medicine (TCBM) interventions, including Chinese herbal formulas, Chinese patent medicines, medicinal botanicals traditionally used in TCM, and their extracts, have increasingly been investigated as potential complementary therapeutic approaches. These interventions have demonstrated effects on multiple ALS-related pathological processes, including oxidative stress, neuroinflammation, and excitotoxicity (Zhang et al., 2014; Suh et al., 2021). However, the available evidence remains fragmented across different pathological processes, intervention types, and experimental systems.

In parallel, selected plant-derived bioactive compounds directly investigated in ALS-specific experimental models have provided complementary pharmacological evidence regarding specific molecular pathways involved in ALS pathology (Mohi-ud-din et al., 2022). Although these compounds are not classified as TCBM interventions, they represent a distinct source of pharmacological evidence that complements the mechanistic understanding of ALS-related pathways. Medicinal botanicals are reported using accepted scientific names, botanical authorship, family designation, and medicinal part or source material whenever reliably identifiable.

Therefore, this review integrates two distinct but complementary streams of pharmacological evidence within a mechanism-oriented framework: evidence from TCBM interventions and evidence from selected plant-derived bioactive compounds directly evaluated in ALS-specific models. This mechanism-oriented framework further considers clinical evidence and translational limitations to provide a clearer basis for future pharmacological research and complementary strategies for ALS.

## 2 Review methodology

To enhance the transparency and reproducibility of this narrative review, a structured literature search was conducted, with study identification and screening reported using a PRISMA-informed approach. Relevant literature was retrieved from PubMed, Web of Science Core Collection, and ScienceDirect through 31 May 2026. The search strategy combined terms related to ALS, TCM and botanical interventions, plant-derived bioactive compounds, and major ALS-related pathological mechanisms. Complete database-specific search strategies, search dates, and corresponding retrieval counts are provided in Supplementary Table S1.

The inclusion criteria were as follows: (a) original publications in English; (b) studies investigating the pharmacological effects or mechanisms of TCBM interventions or plant-derived bioactive compounds and selected translationally relevant derivatives in ALS-specific experimental models; (c) experimental studies elucidating ALS-related pathological mechanisms within predefined pharmacologically relevant domains; and (d) clinical studies directly evaluating these interventions in patients with ALS. The exclusion criteria were as follows: (a) non-English publications; (b) grey literature, conference abstracts, and case reports; (c) duplicate records; (d) studies unrelated to ALS; and (e) studies in which the intervention, experimental design, or outcome data were insufficiently reported for meaningful interpretation. Two researchers independently assessed the selected literature by screening titles and abstracts, followed by full-text assessment when necessary. Disagreements were resolved through discussion with a third reviewer. Duplicate records were identified using title, author, publication year, DOI, and PMID information and were manually verified before screening.

The searches identified 16,993 records, of which 9,301 remained after duplicate removal. Following title and abstract screening, 967 reports underwent full-text assessment, and 198 publications were retained through the structured screening process (Figure 1). Among these, 48 ALS-specific original intervention studies formed the core pharmacological evidence set summarized in Section 4 and Tables 1, 2, while the remaining publications primarily informed the discussion of ALS pathogenesis and clinical evidence. Additional literature on pharmacokinetics, CNS exposure, safety, botanical standardization, biomarkers, and translational considerations was identified through supplementary targeted searches and used as contextual evidence.

**FIGURE 1:**
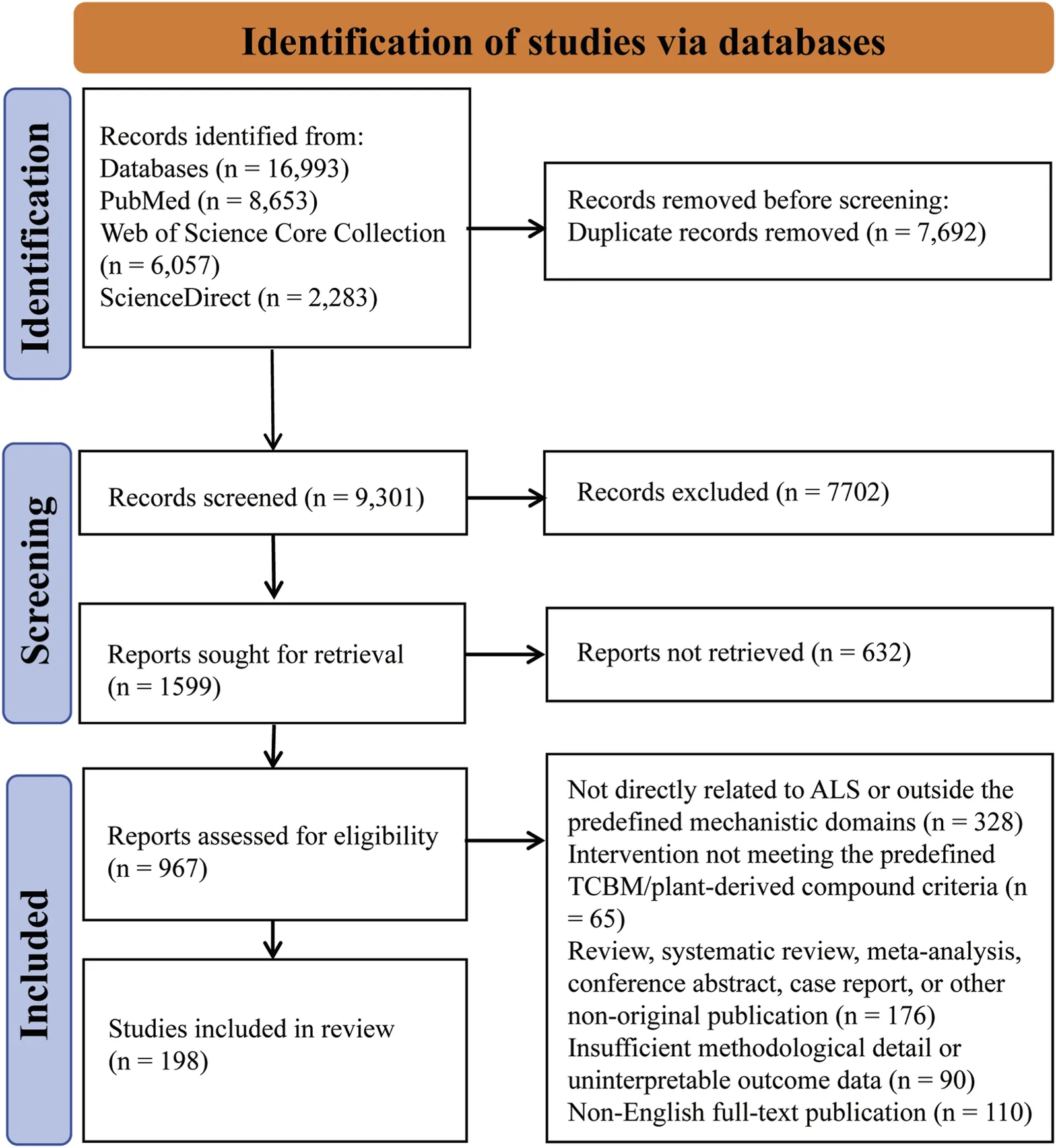
The literature search and screening flowchart. This figure was created with PowerPoint.

**TABLE 1:** Mechanistic pathways of TCBM interventions and plant-derived bioactive compounds in ALS.

Mechanism	Molecular pathway	Intervention	Models	Principal outcomes	Evidence summary/translational status	Evidence level/translational status	Ref
Suppression of glial activation	TLR2/TLR4– NF-κB signaling pathway	Wen-Pi-Tang	*In vivo*: hSOD1-G93A mice	Decrease: GFAP-positive astrocytes, CD11b-positive microglia, HO-1, iNOS. Increase: motor neurons	Single ALS-specific *in vivo* model; no human ALS efficacy evidence	Single-model preclinical evidence; no human ALS efficacy evidence	(Sekiya et al., 2009)
		Huolingshengji Formula	*In vivo*: hSOD1-G93A mice	Decrease: Iba-1, GFAP, TNF-α, COX-2, iNOS. Increase: Bcl-2, NMJ number	Preclinical ALS evidence + Phase II clinical evaluation; primary ALSFRS-R efficacy endpoint was not statistically superior to riluzole	Preclinical evidence + Phase II clinical evaluation; primary ALSFRS-R endpoint not statistically superior to riluzole; further clinical validation required	(Zhou et al., 2018; Liu et al., 2026b)
		Diallyl trisulfide	*In vivo*: hSOD1-G93A mice	Decrease: GFAP, astrocytic activation Increase: HO-1, motor neuron	Single ALS-specific *in vivo* model; no human ALS efficacy evidence	Single-model preclinical evidence; no human ALS efficacy evidence	(Guo et al., 2011)
Inhibition of pro-inflammatory signaling and mediator production		Gamisoyo-San	*In vivo*: female hSOD1-G93A mice	Decrease: CD11b, TLR4, BAX, HO-1, ferritin	Single ALS-specific *in vivo* model; no human ALS efficacy evidence	Single-model preclinical evidence; no human ALS efficacy evidence	(Park and Yang, 2018)
		Jaeumganghwa-Tang	*In vivo*: male hSOD1-G93A mice	Decrease: Iba-1, TLR4, TNF-α, transferrin, ferritin, HO-1, NQO1, BAX.	Single ALS-specific *in vivo* model; no human ALS efficacy evidence	Single-model preclinical evidence; no human ALS efficacy evidence	(Lee and Yang, 2019)
		Genistein	*In vivo*: SOD1-G93A mice	Decrease: Iba-1, GFAP, TLR2, TLR4, NF-κB p65, COX-2, IL-1β, IL-6, TNF-α	Single ALS-specific *in vivo* model; no human ALS efficacy evidence	Single-model preclinical evidence; no human ALS efficacy evidence; Phase I candidacy proposed by the authors	(Zhao et al., 2019)
	NF-κB signaling pathway	Oxymatrine	*In vivo*: female SOD1-G93A mice	Decrease: IBA-1, GFAP, NF-κB, TNF-α, iNOS, IL-1β Increase: CD206, Arg1, IL-4	Single ALS-specific *in vivo* model; no human ALS efficacy evidence	Single-model preclinical evidence; no human ALS efficacy evidence	(Zhang et al., 2021)
Modulation of microglial phenotype-associated responses		Miyako Bidens pilosa extract	*In vivo*: male SOD1-G93A mice *In vitro*: LPS- stimulated BV-2 microglia	Decrease: IFN-γR, TNF-α, IL-1β, IL-6, GFAP, Iba-1, motor neuron loss, LPS-induced TNF-α/IL-1β/IL-6 Increase: SMI32	Single ALS-specific *in vivo* model with complementary inflammatory *in vitro* support; no human ALS efficacy evidence	Multi-model preclinical evidence; no human ALS efficacy evidence	(Kosuge et al., 2020; Tsuruta et al., 2023)
		Allicin	*In vivo*: SOD1-G93A mice	Decrease: Iba-1-positive microglia, CD86-positive M1-like microglia, motor neuron loss Increase: ARG1-positive M2-like microglia	Single ALS-specific *in vivo* model; no significant benefit on disease onset or overall survival; no human ALS efficacy evidence	Single-model preclinical evidence; no human ALS efficacy evidence; no significant benefit on disease onset or overall survival	(Gao et al., 2026)
Suppression of oxidative-stress-generating systems	NADPH oxidase/MAPK signaling pathway	KCHO-1	*In vivo*: female hSOD1-G93A mice *In vitro*: LPS/IFN-γ-activated BV-2 microglia	Decrease: ROS, iNOS, gp91phox, p-ERK1/2; Iba-1, ROS, p-ERK1, p-p38	Single ALS-specific *in vivo* model with complementary inflammatory/oxidative-stress *in vitro* support; no human ALS efficacy evidence	Multi-model preclinical evidence with pharmacological pathway validation; no human ALS efficacy evidence	(Kook et al., 2017)
	NF-κB/iNOS signaling pathway	(−)-Epigallocatechin-3-gallate	*In vivo*: SOD1-G93A mice	Decrease: CD11b-positive activated microglia, iNOS, NF-κB, cleaved caspase-3, cytosolic cytochrome c, cleaved PARP. Increase: PI3K, p-Akt, p-GSK-3	Replicated evidence in the same ALS-specific *in vivo* model across independent studies; no human ALS efficacy evidence	Replicated preclinical evidence; no human ALS data	(Koh et al., 2006; Xu et al., 2006)
Attenuation of lipid peroxidation and radical-mediated oxidative damage		Rosmarinic acid	*In vivo*: hSOD1-G93A mice; *In vitro*: H_2_O_2_-treated SH-SY5Y cells	Decrease: 4-HNE. Increase: ChAT-positive motor neurons	Replicated evidence in the same ALS-specific *in vivo* model, with complementary oxidative-stress *in vitro* support; no human ALS efficacy evidence	Multi-model preclinical evidence with pharmacological pathway validation; no human ALS data	(Shimojo et al., 2010; Seo et al., 2015)
		SK-PC-B70M	*In vivo*: SOD1-G93A mice *In vitro*: H_2_O_2_-treated SH-SY5Y cells	Decrease: MDA, 4-HNE. Increase: ChAT-positive neurons	Single ALS-specific *in vivo* model with complementary oxidative-stress *in vitro* support; no human ALS efficacy evidence	Multi-model preclinical evidence with pharmacological pathway validation; no human ALS data	(Seo et al., 2011)
		EGb 761	*In vivo*: hSOD1-G93A mice	Increase: anterior-horn motor neurons	Single ALS-specific *in vivo* model; proposed antioxidant mechanism was not directly assessed in this ALS model; no human ALS efficacy evidence	Single-model preclinical evidence; no human ALS data	(Ferrante et al., 2001)
Enhancement of endogenous antioxidant defenses	Nrf2/HO-1/GST antioxidant pathway	Shenqi Fuzheng Injection	*In vivo*: male SOD1-G93A mice	Decrease: MDA, GFAP-positive astrocytes Increase: Nrf2, HO-1, GST, SOD, motor neurons	Single ALS-specific *in vivo* model; no human ALS efficacy evidence	Single-model preclinical evidence; no human ALS efficacy evidence	(Sugimoto et al., 2021)
	Akt/Nrf2–GCLC and Akt/STAT3–HSP70 signaling pathways	γ-Oryzanol	*In vivo*: hSOD1-G85R *Drosophila*. *In vitro*: hSOD1-G93A-expressing NSC-34 cells	Decrease: ROS, MDA, oxidized proteins, LDH release, Bax Increase: CAT, GPx, GSH, Bcl-2, p-Akt, Nrf2, GCLC, p-STAT3, HSP70	ALS-specific *Drosophila* and mutant-SOD1 cellular evidence; no mammalian ALS or human efficacy evidence	Multi-model preclinical evidence with pharmacological pathway validation; no mammalian ALS or human efficacy data	(Zhang et al., 2019a)
​	MEK/ERK–HO-1 signaling pathway	Fisetin	*In vivo*: hSOD1-G93A mice and hSOD1-G85R *Drosophila*. *In vitro*: hSOD1-G93A-expressing NSC-34 cells	Decrease: MDA, ROS, hSOD1, detergent-insoluble hSOD1 Increase: CAT, GPx, p-ERK, HO-1, motor neurons	Cross-species ALS-specific preclinical evidence (mouse, *Drosophila*, and cellular mutant-SOD1 models); no human ALS efficacy evidence	Multi-model preclinical evidence; no human ALS efficacy evidence; bioavailability and CNS exposure remain translational limitations	(Wang et al., 2018)
	ALDH1A2/Nrf2/ARE signaling pathway	Apigenin	*In vivo*: SOD1-G93A mice; *In vitro*: SOD1G93A-transfected NSC-34 cells	Decrease: ROS, MDA, 4-HNE, Bax, caspase-3, cytoplasmic TDP-43 Increase: ALDH1A2, SOD, ALDH activity, Bcl-2, Nrf2, HO-1, NQO1	Single ALS genetic context with complementary *in vitro* evidence and target-manipulation support for ALDH1A2; no human ALS efficacy evidence	Multi-model preclinical evidence with target-manipulation support for ALDH1A2; no human ALS efficacy evidence	(Liang et al., 2024)
Modulation of PGC-1α/MFN2-associated mitochondrial homeostasis	PGC-1α/NRF1/TFAM signaling pathway	Honokiol	*In vitro*: SOD1-G93A-expressing NSC-34 cells *In vivo*: male SOD1-G93A mice	Decrease: p62 accumulation Increase: PGC-1α, NRF1, TFAM, OPA1, MFN2, MMP, ATP, mitochondrial complex II/IV activity	Single ALS genetic context with complementary *in vivo* and *in vitro* mechanistic evidence; no human ALS efficacy evidence	Multi-model preclinical evidence with pharmacological support for mitochondrial biogenesis/fusion; no human ALS efficacy evidence	(Zhou et al., 2023)
	SIRT1–AMPK/PGC-1α signaling pathway	Resveratrol	*In vitro*: hSOD1-G93A VSC4.1 motor neuron-like cells; ALS patient-derived BM-MSCs. *In vivo*: SOD1-G93A mice	Decrease: HSF1 acetylation Increase: SIRT1, p-AMPK, NAD, ATP, PGC-1α, MFN2, Hsp25/HSP70 No significant improvement with low-dose dietary resveratrol (25 mg/kg/day)	Replicated preclinical evidence within the SOD1 context, with patient-derived cellular support; lower-dose dietary treatment was ineffective; no human ALS efficacy evidence	Replicated preclinical evidence across multiple models with patient-derived cellular support; no human ALS efficacy evidence	(Markert et al., 2010; Wang et al., 2011; Han et al., 2012; Mancuso et al., 2014; Yun et al., 2018)
	AMPK/PGC-1α/Nrf2/HO-1 signaling pathway	Tetramethylpyrazine nitrone	*In vivo*: SOD1-G93A mice; AAV-TDP-43^M337V^ mouse model	Decrease: ROS, MDA, mutant hSOD1, TDP-43M337V, GFAP/Iba-1 Increase: PGC-1α, Nrf2, HO-1, AMPK-associated signaling	Replicated across mechanistically distinct ALS-specific *in vivo* models (SOD1 and TDP-43) + Phase II clinical evaluation; primary clinical efficacy endpoint was not met.	Multi-model preclinical evidence + Phase II ALS evaluation; primary clinical efficacy endpoint not met	(Huang et al., 2021; Wen et al., 2021; Liu et al., 2025)
Modulation of mitochondrial morphology and organelle homeostasis		Wen-Shen-Jian-Pi prescription	*In vivo*: AR2 mice	Decrease: abnormal mitochondria, autophagosomes	Single ALS-specific *in vivo* model; no human ALS efficacy evidence	Single-model preclinical evidence; no human ALS efficacy evidence	(Gong et al., 2022)
	GRP75-mediated IP3R-VDAC1 signaling pathway	Kaempferol	*In vivo*: symptomatic C9-500 BAC C9ORF72 ALS/FTD mice *In vitro*: C9ORF72 patient-derived iMNs, patient fibroblast- dNs, and C9-500 cortical neurons	Decrease: p-eIF2α, 8-OHdG, Poly (GA) aggregates, GFAP. Increase: GRP75, IP3R–VDAC1 interaction, mitochondrial Ca^2+^ uptake, ATP5J, basal respiration, ATP production, complex I/IV activities	Single C9ORF72 ALS genetic context with patient-derived cellular and *in vivo* support; no human ALS efficacy evidence	Multi-model preclinical evidence; no human ALS efficacy evidence	(Pilotto et al., 2025)
Promotion of UPS-mediated clearance of misfolded proteins	cAMP/PKA signaling	Lycorine	*In vivo*: TDP-43A315T transgenic mice; TDP-43A315T *Caenorhabditis elegans* *In vitro*: TDP-43A315T-expressing HeLa cells	Decrease: TDP-43A315T and UPS reporter substrates Increase: UPS-mediated clearance	Cross-species TDP-43 ALS/proteinopathy evidence (mouse, *C. elegans*, and cellular models); no human ALS efficacy evidence	Cross-species preclinical evidence; no human ALS efficacy evidence	(Wen et al., 2024)
		Baicalein	*In vivo*: SOD1-G93A mice; *In vitro*: SOD1/G93A-transfected NSC-34 cells	Decrease: soluble/insoluble misfolded SOD1 Increase: K48-linked ubiquitinated proteins; motor-neuron; NMJs	Single ALS genetic context with complementary *in vivo* and *in vitro* evidence; no human ALS efficacy evidence	Multi-model preclinical evidence; no human ALS efficacy evidence	(Ting et al., 2025)
Modulation of autophagy–lysosomal pathways and aggregate clearance		Achyranthes–Gastrodia–Chaenomeles formulation	*In vivo*: male hSOD1-G93A mice	Decrease: p62, LC3B, TGF-β, SMAD2, HO-1, ferritin, Bax Increase: ChAT-positive and Nissl-positive motor neurons	Single ALS-specific *in vivo* model; no human ALS efficacy evidence	Single-model preclinical evidence; no human ALS efficacy evidence	(Yang, 2025)
		Pseudocydonia sinensis extract	*In vivo*: male hSOD1-G93A mice	Decrease: p62, LC3B, p-mTOR, SMAD2, TLR4, IL-1β, GFAP, Iba-1, HO-1, Bax, ferritin, transferrin Increase: ChAT-positive and Nissl-positive motor neurons	Single ALS-specific *in vivo* model; no human ALS efficacy evidence	Single-model preclinical evidence; no human ALS efficacy evidence	(Yang and Lee, 2023)
		Paeonia lactiflora extract	*In vivo*: male hSOD1-G93A mice	Decrease: p62, LC3B, SMAD2, ferritin, HO-1, transferrin, 4-HNE, Iba-1, GFAP. Increase: ChAT-positive motor neurons	Single ALS-specific *in vivo* model; no human ALS efficacy evidence	Single-model preclinical evidence; no human ALS efficacy evidence	(Yang and Lee, 2025)
		Pu-erh tea extract	*In vitro*: SK-N-SH cells expressing WT or R521C FUS/TLS	Decrease: FUS/TLS, EWS, TAF15, cytoplasmic R521C FUS/TLS, FUS-positive stress-granule incorporation Increase: lysosome-dependent FET-protein clearance	*In vitro* ALS-associated FUS proteinopathy evidence with pharmacological inhibitor support for lysosome-dependent clearance; no *in vivo* or human ALS evidence	Single-model preclinical evidence; no *in vivo* or human ALS evidence	(Yu et al., 2014)
​	mTOR/p70S6K signaling pathway	Berberine	*In vitro*: N2a cells expressing aggregation-prone GFP-TDP-25	Decrease: insoluble TDP-43, TDP-43 aggregates, p-mTOR, p-p70S6K Increase: LC3-II, autophagosome formation	*In vitro* ALS-associated TDP-43 proteinopathy evidence with pharmacological autophagy-inhibitor support; no ALS animal or human evidence	Single-model preclinical evidence; no ALS animal or human evidence	(Chang et al., 2016)
Enhancement of chaperone responses and attenuation of ER stress		Celastrol	*In vivo*: G93A-SOD1 transgenic mice	Decrease: TNF-α, iNOS, CD40, GFAP. Increase: HSP70	Single ALS-specific *in vivo* model; no human ALS efficacy evidence	Single-model preclinical evidence; no human ALS efficacy evidence	(Kiaei et al., 2005)
Targeting glutamate excitotoxicity and calcium dysregulation		Nordihydroguaiaretic acid	*In vitro*: MN-1 neural cells; *In vivo*: SOD1-G93A mice	Increase: high-affinity glutamate uptake, EAAT2-sensitive glutamate uptake, spinal glutamate uptake	Single ALS-specific *in vivo* model with complementary glutamate-uptake *in vitro* support; target engagement was transient and no survival benefit was observed; no human ALS efficacy evidence	Multi-model preclinical evidence; no human ALS efficacy evidence	(Boston-Howes et al., 2008)
Preservation of NMJ integrity and muscle–motor neuron connectivity	Wnt/β-catenin/GSK-3β-associated signaling pathway	Bojungikgi-Tang	*In vivo*: hSOD1-G93A mice	Decrease: TGF-β, Prox1, β-catenin Increase: p-GSK-3β (Ser9)	Single ALS-specific *in vivo* model; no human ALS efficacy evidence	Single-model preclinical evidence; no human ALS efficacy evidence	(Cai M. et al., 2019)
		Four-botanical herbal extract	*In vivo*: male hSOD1-G93A mice	Decrease: GFAP, CD11b, TNF-α, HO-1, NQO1, Bax, ferritin, p62, LC3B Increase: α-bungarotoxin-positive NMJs	Single ALS-specific *in vivo* model; no human ALS efficacy evidence	Single-model preclinical evidence; no human ALS efficacy evidence	(Lee et al., 2021)
		Protocatechuic acid	*In vivo*: hSOD1-G93A mice	Decrease: GFAP, Iba-1, NMJ denervation Increase: NMJ area, α-BTx/VAChT-associated NMJ innervation, α-motor neurons	Single ALS-specific *in vivo* model; no human ALS efficacy evidence	Single-model preclinical evidence; no human ALS efficacy evidence	(Koza et al., 2020)
Enhancement of neurotrophic signaling and motor neuron support		7,8-Dihydroxyflavone	*In vivo*: male SOD1-G93A mice	Increase: total motor neuron count, small motor neuron count	Single ALS-specific *in vivo* model; no human ALS efficacy evidence	Single-model preclinical evidence; no human ALS efficacy evidence	(Korkmaz et al., 2014)
	LPA1 receptor signaling	Gintonin	*In vivo*: male G93A-SOD1 mice	Increase: LPA1 receptor, BDNF-positive cells, ChAT-positive cells, Nissl-positive neurons, NeuN-positive neurons	Single ALS-specific *in vivo* model with reported survival benefit; no human ALS efficacy evidence	Single-model preclinical evidence with long-term oral treatment and survival benefit; no human ALS efficacy evidence	(Nam et al., 2021)

4-HNE, 4-hydroxynonenal; AAV, adeno-associated virus; ALS, amyotrophic lateral sclerosis; ALSFRS-R, Amyotrophic Lateral Sclerosis Functional Rating Scale-Revised; AMPK, AMP-activated protein kinase; ATP, adenosine triphosphate; BM-MSCs, bone marrow-derived mesenchymal stem cells; CNS, central nervous system; COX-2, cyclooxygenase-2; EAAT2, excitatory amino acid transporter 2; ER, endoplasmic reticulum; FTD, frontotemporal dementia; GSH, glutathione; GST, glutathione S-transferase; HO-1, heme oxygenase-1; IL, interleukin; iNOS, inducible nitric oxide synthase; LPS, lipopolysaccharide; MAPK, mitogen-activated protein kinase; MDA, malondialdehyde; NADPH, nicotinamide adenine dinucleotide phosphate; NF-κB, nuclear factor kappa B; NMJ, neuromuscular junction; PGC-1α, peroxisome proliferator-activated receptor gamma coactivator-1, alpha; PKA, protein kinase A; ROS, reactive oxygen species; TGF-β, transforming growth factor beta; TNF-α, tumor necrosis factor alpha; UPS, ubiquitin–proteasome system.

**TABLE 2:** Botanical composition and dosing characteristics of TCBM interventions and plant-derived bioactive compounds in ALS studies.

Intervention	Composition	Extract type	Dosage range	Minimal active concentration	Treatment duration	Ref
Wen-Pi-Tang	Aqueous extract of *Rheum officinale* Baill. [Polygonaceae; Rhei Rhizoma], *Panax ginseng* C.A.Mey. [Araliaceae; Ginseng Radix], *Aconitum japonicum* Thunb. [Ranunculaceae; Aconiti Tuber], *Zingiber officinale* Roscoe [Zingiberaceae; Zingiberis Rhizoma], and *Glycyrrhiza glabra* L. [Fabaceae; Glycyrrhizae Radix] (15:3:9:3:5)	Aqueous extract	100 or 200 mg/kg/day, p.o	200 mg/kg/day	7–16 weeks of age (∼9 weeks)	(Sekiya et al., 2009)
Huolingshengji Formula	*Astragalus mongholicus* Bunge [Fabaceae; Astragali Radix], Epimedii Herba (source taxon not specified in the ALS study), *Rehmannia glutinosa* (Gaertn.) DC. [Orobanchaceae; Rehmanniae Radix], *Cornus officinalis* Siebold & Zucc. [Cornaceae; Corni Fructus], *Atractylodes macrocephala* Koidz. [Asteraceae], and Poria (sclerotium; source taxon not specified) (5:6:4:5:3:3)	Formula granule	3, 4.5, or 6 g/kg/day, p.o. by gavage; post-onset/mechanistic cohorts: 4.5 g/kg/day	4.5 g/kg/day	P57-death; post-onset: onset-death; mechanistic: P57-P110	(Zhou et al., 2018; Liu et al., 2026b)
Diallyl trisulfide	Garlic-oil organosulfur compound from *Allium sativum* L. [Amaryllidaceae]	Pure compound	80 mg/kg/day, p.o., once daily	Not established	Onset-endpoint; pathology: 20 days	(Guo et al., 2011)
Gamisoyo-San	*Bupleurum chinense* DC. [Apiaceae], *Angelica sinensis* (Oliv.) Diels [Apiaceae], *Paeonia lactiflora* Pall. [Paeoniaceae], *Atractylodes macrocephala* Koidz. [Asteraceae], Poria (sclerotium; source taxon not specified in the ALS study), fresh *Zingiber officinale* Roscoe [Zingiberaceae], *Paeonia suffruticosa* Andrews [Paeoniaceae], *Gardenia jasminoides* J.Ellis [Rubiaceae], *Mentha haplocalyx* Briq. [Lamiaceae], and *Glycyrrhiza uralensis* Fisch. Ex C. [Fabaceae]	Commercial formula	1 g/kg/day, p.o., once daily	Not established	6 weeks (8–14 weeks of age)	(Park and Yang, 2018)
Jaeumganghwa-Tang	Traditional 12-botanical formula; individual source taxa were not specified in the ALS study	Commercial formula	1 g/kg/day, p.o., once daily	Not established	6 weeks (8–14 weeks of age)	(Lee and Yang, 2019)
Genistein	Plant-derived isoflavone occurring in soybean, *Glycine* max (L.) Merr. [Fabaceae], and other legumes	Pure compound	16 mg/kg, p.o., twice daily (32 mg/kg/day)	Not established	8–16 weeks of age (∼8 weeks)	(Zhao et al., 2019)
Oxymatrine	Quinolizidine alkaloid associated with the root of *Sophora flavescens* Aiton [Fabaceae]	Pure compound	100 mg/kg/day, i.p., once daily	Not established	P55-end stage	(Zhang et al., 2021)
Miyako Bidens pilosa extract	Extract of Miyako Island *Bidens pilosa* L. var. *radiata* Scherff [Asteraceae]	Commercial botanical preparation	*In vivo*: 2 g/kg/day, p.o. by gavage, 5 days/week. *In vitro*: 100 or 1,000 μg/mL Miyako *Bidens pilosa* extract with 1 μg/mL LPS.	*In vitro*: 100 μg/mL; *In vivo*: not established	Kosuge: onset-death; mechanistic: 3 weeks. Tsuruta: 1 week *in vivo*; 4 h *in vitro*	(Kosuge et al., 2020; Tsuruta et al., 2023)
Allicin	Garlic-derived organosulfur compound from *Allium sativum* L. [Amaryllidaceae]	Pure compound	10 mg/kg, p.o. by gavage, every other day	Not established	P60-endpoint; tissue analysis at P110	(Gao et al., 2026)
KCHO-1	30% ethanol extract of nine botanicals: *Curcuma longa* L. [Zingiberaceae], *Salvia miltiorrhiza* Bunge [Lamiaceae], *Gastrodia elata* Blume [Orchidaceae], *Pseudocydonia sinensis* (Dum.Cours.) C.K.Schneid. [Rosaceae; reported as *Chaenomeles sinensis*], *Polygala tenuifolia* Willd. [Polygalaceae], *Paeonia japonica* (Makino) Miyabe & Takeda [Paeoniaceae], *Glycyrrhiza uralensis* Fisch. ex DC. [Fabaceae], *Atractylodes japonica* Koidz. ex Kitam. [Asteraceae], and processed *Aconitum carmichaelii* Debeaux [Ranunculaceae] (4:4:4:2:2:2:2:2:1)	30% ethanol extract	*In vivo*: 250 mg/kg/day, p.o. *In vitro*: 3.1–100 μg/mL KCHO-1 with LPS + IFN-γ (10 ng/mL each)	*In vitro*: 50 μg/mL; *In* *vivo*: not established	*In vivo*: 4 weeks (P91-P119); *In vitro*: 24 h	(Kook et al., 2017)
(−)-Epigallocatechin-3-gallate	Major tea catechin from *Camellia sinensis* (L.) Kuntze [Theaceae]	Pure compound	Xu et al.: 10 mg/kg/day, intraoral. Koh et al.: 1.5, 2.9, or 5.8 mg/kg/day, intraoral, once daily	2.9 mg/kg/day	Xu: P42-death; Koh: P60-endpoint	(Koh et al., 2006; Xu et al., 2006)
Rosmarinic acid	Plant-derived phenolic acid occurring in multiple Lamiaceae species; *Perilla frutescens* (L.) Britton [Lamiaceae] is one reported medicinal source	Pure compound	Shimojo et al.: 0.13 mg/kg, i.p., twice weekly. Seo et al.: 30 or 400 mg/kg/day, p.o. *In vitro*: 15 or 30 μg/mL	*In vivo*: 0.13 mg/kg; *In vitro*: 15 μg/mL	Shimojo: P63-endpoint; Seo: 9 weeks (7–16 weeks)	(Shimojo et al., 2010; Seo et al., 2015)
SK-PC-B70M	Oleanolic-glycoside saponin fraction isolated from the root of *Pulsatilla cernua* var. *koreana* (Y.Yabe ex Nakai) Y.N.Lee [Ranunculaceae]	Saponin-enriched fraction	*In vivo*: 100 or 400 mg/kg/day in chow. *In vitro*: 100 μg/mL with 800 μM H_2_O_2_	100 mg/kg/day (behavior); 400 mg/kg/day (4-HNE); 100 μg/mL *in* *vitro*	*In vivo*: ∼8–16 weeks of age; *In vitro*: 24 h	(Seo et al., 2011)
EGb 761	Standardized leaf extract of *Ginkgo biloba* L. [Ginkgoaceae] (EGb 761)	Standardized leaf extract	0.022% or 0.045% in diet (approximately 200 or 400 mg/kg/day)	0.022% diet (∼200 mg/kg/day)	P21-endpoint	(Ferrante et al., 2001)
Shenqi Fuzheng Injection	*Codonopsis pilosula* (Franch.) Nannf. [Campanulaceae; Codonopsis Radix] + Astragali Radix (pharmacopoeial source: *Astragalus mongholicus* Bunge [Fabaceae]) (1:1; 250 mL Shenqi Fuzheng Injection contains 10 g of each crude drug)	Injectable botanical preparation	40 mL/kg, i.p., 3 times/week	Not established	P87-neurological score 4; pathology to P130	(Sugimoto et al., 2021)
γ-Oryzanol	Mixture of ferulic-acid steryl/triterpenyl esters associated with rice bran/oil from *Oryza sativa* L. [Poaceae]	Commercial compound mixture	*Drosophila*: 15, 25, or 50 μM in food. Cells: 0.1–5 μM	15 μM (survival); 1 μM (cells)	*Drosophila*: lifelong from day 1; mechanistic: 28 days; cells: 24 h	(Zhang et al., 2019a)
Fisetin	Plant-derived flavonoid occurring in multiple botanical sources	Pure compound	Mice: 9 mg/kg/day by gavage. *Drosophila*: 1–300 μM in food. Cells: 10 μM	*Drosophila*: 100 μM; cells: 10 μM; mice: not established	Mice: P60-endpoint; *Drosophila*: 28 days; cells: 4–24 h	(Wang et al., 2018)
Apigenin	Plant-derived flavonoid	Pure compound	*In vivo*: 40 or 80 mg/kg/day, p.o., once daily. *In vitro*: 1 μM	40 mg/kg/day; 1 μM *in vitro*	*In vivo*: throughout study; *In vitro*: 24 h	(Liang et al., 2024)
Honokiol	Biphenolic neolignan associated with the bark, stem, and root of *Magnolia officinalis* Rehder & E.H.Wilson [Magnoliaceae]	Pure compound	Cells: 3 or 10 μM. Mice: 10, 30, or 90 mg/kg, i.p., once daily	30 mg/kg/day *in vivo*; 3 μM *in vitro*	Cells: 12–24 h; mice: onset-end stage; mechanistic: 11–18 weeks	(Zhou et al., 2023)
Resveratrol	Plant-derived stilbenoid occurring in multiple botanical sources; Japanese knotweed, *Reynoutria japonica* Houtt. [Polygonaceae; syn. *Polygonum cuspidatum* Siebold & Zucc.], and grapes are reported sources	Pure compound	*In vitro*: 10 μM (VSC4.1 cells) or 1 μM (ALS patient-derived mesenchymal stem cells). *In vivo*: 160 mg/kg/day dietary; 20 mg/kg i.p. twice weekly; or 25 mg/kg/day dietary	20 mg/kg i.p. twice weekly; 160 mg/kg/day dietary; cells: 1–10 microM	Cells: 12–48 h; mice: 8/12 weeks-endpoint, P60-death, or P47-endpoint by regimen	(Markert et al., 2010; Wang et al., 2011; Han et al., 2012; Mancuso et al., 2014; Yun et al., 2018)
Tetramethylpyrazine nitrone	Synthetic nitrone derivative of tetramethylpyrazine (TMP)	Synthetic derivative	SOD1-G93A: 10, 30, or 60 mg/kg twice daily, i.p. or i.g. TDP-43M337V: 30 mg/kg by oral gavage twice daily	10 mg/kg twice daily	SOD1-G93A: 4–6 weeks from onset; TDP-43M337V: study-specific	(Huang et al., 2021; Wen et al., 2021; Liu et al., 2025)
Wen-Shen-Jian-Pi prescription	Study-reported seven-drug formula: Ginseng Radix (15 g), Astragali Radix (15 g), Cistanches Herba (30 g), Atractylodis Macrocephalae Rhizoma (15 g), Poria (15 g), Glycyrrhizae Radix (10 g), and Epimedii Herba (25 g). (The primary ALS study did not provide species-level botanical authentication for these crude drugs; therefore, no source taxa are assigned here.)	Aqueous decoction extract	15, 30, or 45 mg/kg/day, p.o. by gavage, once daily	15 mg/kg/day	12 weeks (from 17 weeks of age)	(Gong et al., 2022)
Kaempferol	Dietary flavonol occurring in multiple botanical sources; *Crocus sativus* L. [Iridaceae] is one reported source	Pure compound	*In vivo*: 10 mg/kg, i.p. *In vitro*: 10 μg/mL	Not established	*In vivo*: daily 2 weeks, then every other day to P240; *In vitro*: 2–48 h	(Pilotto et al., 2025)
Lycorine	Amaryllidaceae alkaloid reported from bulbs of *Lycoris radiata* (L’Hér.) Herb. [Amaryllidaceae]	Pure compound	Cells: 10 μM. *C. elegans*: 25–75 μM. Mice: 10 mg/kg, i.p., once daily	Not established	Cells: 12–24 h; *C. elegans*: study period; mice: 4 weeks (P90 cohort) or P60-death	(Wen et al., 2024)
Baicalein	Flavonoid associated with the root of *Scutellaria baicalensis* Georgi [Lamiaceae]	Pure compound	*In vitro*: 50 or 100 μM. *In vivo*: 13.6 mg/kg/day, i.p	Not established	*In vitro*: 24 h; *In vivo*: 2–4.5 months; survival: 2 months-death	(Ting et al., 2025)
Achyranthes–Gastrodia–Chaenomeles formulation	Extracts of *Achyranthes bidentata* Blume [Amaranthaceae], *Gastrodia elata* Blume [Orchidaceae], and *Pseudocydonia sinensis* (Dum.Cours.) C.K.Schneid. [Rosaceae; reported as *Chaenomeles sinensis*] (1:1:1)	Combined botanical extract	1,000 mg/kg/day, p.o., once daily	Not established	6 weeks	(Yang, 2025)
Pseudocydonia sinensis extract	Aqueous extract of *Pseudocydonia sinensis* (Dum.Cours.) C.K.Schneid. [Rosaceae]	Aqueous extract	1,000 mg/kg/day, p.o., once daily	Not established	6 weeks	(Yang and Lee, 2023)
Paeonia lactiflora extract	Aqueous extract of *Paeonia lactiflora* Pall. [Paeoniaceae]	Aqueous extract	300 mg/kg/day, p.o., once daily	Not established	6 weeks	(Yang and Lee, 2025)
Pu-erh tea extract	Fermented Pu-erh tea extract	Fermented tea extract	50–250 μg/mL; principal mechanistic experiments used 200 μg/mL	Not established	24 h	(Yu et al., 2014)
Berberine	Isoquinoline alkaloid; *Coptis chinensis* Franch. [Ranunculaceae] is a recognized medicinal source	Pure compound	10, 20, or 30 μg/mL; 20 μg/mL in the 3-MA validation experiment	Not established	24 h	(Chang et al., 2016)
Celastrol	Quinone methide triterpenoid associated with the root bark of *Tripterygium wilfordii* Hook.f. [Celastraceae]	Pure compound	2 or 8 mg/kg/day, orally via diet.	2 mg/kg/day	P30-endpoint	(Kiaei et al., 2005)
Nordihydroguaiaretic acid (NDGA)	Plant-derived lignan abundant in *Larrea tridentata* (Sessé & Moc. ex DC.) Coville [Zygophyllaceae]	Pure compound	*In vitro*: 1–10 μM (4 μM commonly used). *In vivo*: 10 mg/10-day sustained-release pellet, s.c	Not established	*In vivo*: onset-endpoint; pellet replaced every 10 days; *In vitro*: assay-dependent	(Boston-Howes et al., 2008)
Bojungikgi-Tang	Study-reported multi-botanical preparation containing Angelicae Gigantis Radix, Astragali Radix, Atractylodis Rhizoma, Bupleuri Radix, Cimicifugae Rhizoma, Citri Unshii Pericarpium, Ginseng Radix Alba, and Glycyrrhizae Radix	Commercial formula	1 g/kg/day, p.o., once daily	Not established	6 weeks (from 8 weeks of age)	(Cai M. et al., 2019)
Four-botanical herbal extract	Aqueous 1:1:1:1 extract of *Glycyrrhiza uralensis* Fisch.ex DC [Fabaceae], *Atractylodes macrocephala* Koidz. [Asteraceae], *Panax ginseng* C.A.Mey. [Araliaceae], and *Astragalus mongholicus* Bunge [Fabaceae] (reported as *Astragalus membranaceus* in the original study)	Aqueous extract	1 g/kg/day, p.o., once daily	Not established	6 weeks (from 8 weeks of age)	(Lee et al., 2021)
Protocatechuic acid	Plant-derived phenolic acid/anthocyanin metabolite occurring in multiple botanical sources	Pure compound	50 or 100 mg/kg, p.o. by gavage, 5 days/week	50 mg/kg	Onset (∼P90)-end stage, 5 days/week	(Koza et al., 2020)
7,8-Dihydroxyflavone	Flavonoid isolated from *Oroxylum indicum (L.) Kurz [*Bignoniaceae*]*	Pure compound	5 mg/kg, i.p., 3 days/week	Not established	1 month of age-P105	(Korkmaz et al., 2014)
Gintonin	Gintonin-enriched glycolipoprotein fraction from *Panax ginseng* C.A.Mey. [Araliaceae]	Gintonin-enriched fraction	50 or 100 mg/kg/day, p.o	50 mg/kg/day	11 weeks (7–18 weeks of age)	(Nam et al., 2021)

ALS, amyotrophic lateral sclerosis; IFN-γ, interferon gamma; LPS, lipopolysaccharide; P, postnatal day; TCBM, traditional Chinese botanical medicine; i.g., intragastric administration; i.p., intraperitoneal administration; p.o., oral administration; s.c., subcutaneous administration.

## 3 Pathogenesis

The pathogenesis of ALS involves multiple molecular and cellular processes, including genetic abnormalities and pathogenic protein misfolding, oxidative stress and mitochondrial dysfunction, impaired proteostasis and endoplasmic reticulum (ER) stress, neuroinflammation, glutamate excitotoxicity, and neuromuscular junction (NMJ) dysfunction. The major pathological processes discussed in this review are summarized in Figure 2.

**FIGURE 2:**
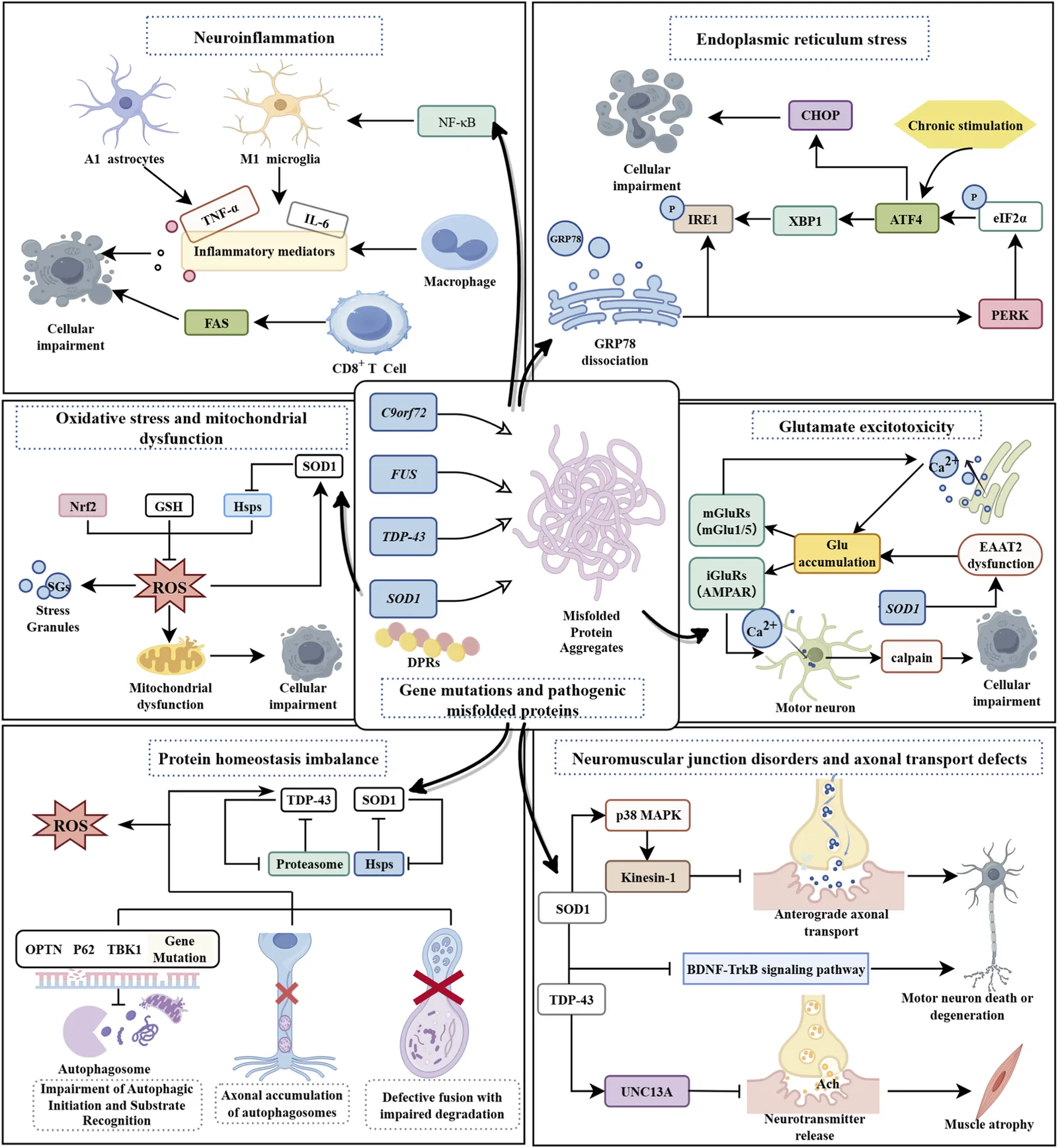
Pathological Mechanisms of ALS. The pathological mechanisms of ALS involve gene mutations and the formation of pathogenic misfolded proteins, neuroinflammation, endoplasmic reticulum stress, oxidative stress and mitochondrial dysfunction, glutamate excitotoxicity, protein homeostasis imbalance, neuromuscular junction dysfunction, and axonal transport defects. The aggregation of misfolded proteins, particularly involving SOD1, TDP-43, FUS, and C9orf72-related products, is a core pathological feature of ALS and is more closely associated with other pathological processes. When these pathological mechanisms are disrupted or impaired, they respond through related pathways, leading to motor neuron death or degeneration and muscle atrophy. NF-κB, Nuclear factor kappa-B; TNF-α, Tumor Necrosis factor-Alpha; IL-6, Interleukin 6; CD8^+^ T cell, Cluster of Differentiation 8 positive T lymphocyte; Nrf2, Nuclear factor erythroid 2-related factor 2; GSH, Glutathione; Hsp, Heat shock protein; ROS, Reactive oxygen species; C9orf72, Chromosome 9 open reading frame 72; FUS, Fused in sarcoma; TDP-43, TAR DNA-binding protein 43; SOD1, Superoxide dismutase 1; DPRs, Dipeptide-repeat proteins; CHOP, C/EBP Homologous Protein; IRE1, Inositol-requiring enzyme 1α; PERK, Protein kinase RNA-like endoplasmic reticulum kinase; ATF4, Activating transcription factor 4; XBP1, X-box-binding protein 1; eIF2α, Eukaryotic translation-initiation factor 2α; GRP78, Glucose-regulated protein 78; Glu, glutamate; iGluRs, ionotropic glutamate receptors; mGluRs, metabotropic glutamate receptors; EAAT2, Excitatory amino acid transporter 2; p38 MAPK, p38 mitogen-activated protein kinase; BDNF, Brain-derived neurotrophic factor; TrkB, Tropomyosin receptor kinase B; UNC13A, Unc-13 homolog A; Ach, Acetylcholine. This figure was created with Figdraw (ID: SIOYOb3bb5).

### 3.1 Gene mutations and pathogenic misfolded proteins

The genetic risk factors for most sporadic ALS remain unclear. *SOD1*, TAR DNA-binding protein gene (*TARDBP*) encoding TAR DNA-binding protein 43 (TDP-43), fused in sarcoma (*FUS*), and chromosome 9 open reading frame 72 (*C9orf72*) currently represent the most extensively described major disease-causing genes in ALS.

*SOD1* encodes copper/zinc superoxide dismutase, a highly stable antioxidant enzyme located mainly in the cytoplasm and mitochondria, where it limits superoxide-mediated oxidative injury (Peggion et al., 2022). Pathogenic *SOD1* mutations alter protein conformation and function, promoting oxidative stress and mitochondrial dysfunction (Wang X.-X. et al., 2024). Increased free radicals and oxidative damage have been detected in serum, cerebrospinal fluid (CSF), and urine from patients with *SOD1*-mutant ALS (Zarei et al., 2015). Mutant SOD1 can also accumulate at mitochondrial membranes and within the intermembrane space, thereby disrupting mitochondrial function (Boillée et al., 2006; Sheng et al., 2013). In addition, impaired ubiquitin-proteasome system (UPS) activity limits clearance of misfolded proteins, while mutant SOD1 can interact with derlin-1 and inhibit ER-associated degradation, aggravating ER stress, proteostatic failure, and apoptotic signaling (Kikuchi et al., 2006; Zhao et al., 2022).

*TARDBP* encodes TDP-43, a predominantly nuclear DNA/RNA-binding protein involved in RNA splicing, gene regulation, and DNA damage repair (Baralle et al., 2013; Heyburn and Moussa, 2017; Mitra et al., 2019). Pathogenic *TARDBP* mutations promote nuclear depletion and cytoplasmic accumulation of TDP-43, producing inclusions that represent a major pathological hallmark of ALS (Prasad et al., 2019). Approximately 97% of fALS and sALS patients exhibit TDP-43-positive inclusions in the spinal cord or motor cortex (Konopka et al., 2020). Abnormally aggregated TDP-43 interacts closely with cellular stress responses and is recruited into newly formed stress granules. Stress granules may exhibit cytotoxicity and can in turn promote the pathological progression of TDP-43 (Nishimoto et al., 2010). Concurrently, TDP-43 dysfunction impairs DNA repair, and the resulting accumulation of DNA damage further induces the formation of additional cytoplasmic inclusions, ultimately triggering neurodegeneration (Konopka et al., 2020). Furthermore, abnormal TDP-43 aggregation is directly associated with elevated oxidative stress levels and can induce ER stress, leading to apoptosis (Zhao et al., 2022). Its toxic effects also include disrupting mitochondrial structure and function, triggering neuroinflammation, and causing abnormalities in other cellular pathways such as autophagy, collectively leading to motor neuron death (Dafinca et al., 2021).

*FUS* encodes another DNA/RNA-binding protein of the FET family and participates in gene regulation and DNA damage repair (Ratti and Buratti, 2016). Under physiological conditions, FUS is mainly nuclear, whereas ALS-associated mutations promote its cytoplasmic mislocalization and accumulation in lower motor neurons, causing both toxic gain of function and loss of normal nuclear activity (Daigle et al., 2013). Increased FUS expression and mislocalization have also been observed in astrocytes of SOD1-G93A mice and were associated with neuronal injury, suggesting a contribution to non-cell-autonomous toxicity (Li et al., 2016). Mutant FUS can disrupt nonsense-mediated decay and protein translation, accumulate in axons, activate local integrated stress responses, and impair synaptic protein synthesis (Kamelgarn et al., 2018; López-Erauskin et al., 2018). It can also reduce mitochondrial membrane potential and respiratory capacity, linking FUS pathology to mitochondrial dysfunction (Tsai et al., 2020).

The G4C2 hexanucleotide repeat expansion in the non-coding region of *C9orf72* is the most common genetic cause of ALS in populations of European ancestry (DeJesus-Hernandez et al., 2011). Healthy individuals generally carry relatively few repeats, whereas affected patients may harbor hundreds to thousands, although the exact pathogenic threshold remains uncertain (Beckers et al., 2021). C9orf72 protein contributes to autophagy-lysosomal regulation and lipid metabolism in the central nervous system (CNS) (Pang and Hu, 2021). Disease mechanisms involve both toxic gain of function and loss of function. Expanded repeats generate repeat-rich RNAs and, through repeat-associated non-ATG translation, five dipeptide repeat proteins: poly-GA, poly-GR, poly-PR, poly-PA, and poly-GP (Babić Leko et al., 2019; Wang et al., 2023). Poly-GR, poly-PR, and poly-GA are particularly neurotoxic and can impair DNA damage responses and repair pathways (Kok et al., 2021). Poly-GR additionally interacts with mitochondrial ribosomal proteins, promoting mitochondrial dysfunction, reactive oxygen species (ROS) production, and further DNA damage (Lopez-Gonzalez et al., 2016). Reduced C9orf72 expression may further impair normal cellular functions and enhance dipeptide-repeat accumulation, suggesting that loss-of-function and toxic gain-of-function mechanisms may act synergistically to drive neurodegeneration (Zhu et al., 2020).

### 3.2 Oxidative stress and mitochondrial dysfunction

In the pathogenesis of ALS, oxidative stress and mitochondrial dysfunction represent two mutually reinforcing pathological processes that jointly drive the progressive degeneration of MNs. As the cell’s powerhouse, mitochondria generate adenosine triphosphate (ATP) through oxidative phosphorylation and serve as a central organelle regulating apoptosis, calcium homeostasis, and metabolism (Iwata and Vanderhaeghen, 2021). During electron transport and ATP synthesis in mitochondria, a small number of leaked electrons combine with oxygen molecules to generate ROS, which serve as normal metabolic byproducts (Van Der Bliek et al., 2017). Under physiological conditions, these ROS can be effectively cleared by the antioxidant system and participate in cellular signaling. However, when the production and clearance of intracellular ROS become imbalanced, leading to excessive accumulation of ROS that subsequently damages biomolecules such as proteins, lipids, and DNA, this process is termed oxidative stress.

In ALS patients, oxidative stress levels are significantly elevated. Evidence indicates that in both ALS patients and disease models such as SOD1-G93A transgenic mice, substantial increases in multiple oxidative stress markers can be detected, including lipid peroxidation damage and stress-related proteins (Siciliano et al., 2007). This phenomenon is closely associated with the dysfunction of the endogenous antioxidant defense system. On one hand, nuclear factor erythroid 2-related factor 2 (Nrf2), as the primary regulator of numerous antioxidant enzymes, protects cells from oxidative stress. Studies have revealed that both the transcriptional and protein levels of Nrf2 are significantly downregulated in MNs of ALS patients (Sarlette et al., 2008). On the other hand, metabolic imbalance in the glutathione (GSH) antioxidant system disrupts key processes in ROS clearance and oxidative damage repair (Kim, 2021). Preclinical studies have demonstrated that alleviating cellular oxidative stress by enhancing GSH synthesis and increasing Nrf2 expression exerts neuroprotective effects on MNs in ALS fruit fly models (Zhang et al., 2019b). Additionally, abnormal protein aggregates such as FUS and SOD1 promote ROS production in ALS patients by inducing the depletion of cellular chaperones like heat shock protein 70 (Hsp70) (Mogk et al., 2018). Oxidative stress contributes to ALS pathogenesis through multiple direct and indirect mechanisms. First, excessive ROS directly damage mRNA sequences responsible for protein synthesis and electron transport chain function within mitochondria, inducing the production of damaged proteins (Barber and Shaw, 2010). Secondly, ROS can induce abnormal post-translational modifications in RNA-binding proteins such as TDP-43 and FUS. These modifications promote pathological liquid–liquid phase separation to form insoluble aggregates, ultimately leading to neurodegenerative disease. This phenomenon of abnormal post-translational modifications has been observed in both sALS and fALS (Harley et al., 2021).

Neurons possess high energy demands, and the maintenance of their physiological functions is highly dependent on the dynamic functional network of mitochondria. Therefore, mitochondrial dysfunction is often regarded as a key phenomenon in the pathophysiology of ALS (Smith et al., 2019). Research has revealed that ALS patients exhibit morphological changes in motor neuron mitochondria, including abnormal cellular distribution, swelling, vacuolation, and disruption of cristae structures (Xu et al., 2004). Furthermore, in TDP-43 and FUS-induced ALS *Drosophila* models, mitochondrial fission in muscles and MNs is significantly enhanced, suggesting that normal mitochondrial dynamics are impaired (Altanbyek et al., 2016). In bioenergetics, reduced mitochondrial DNA copy numbers and significantly diminished activity of mitochondrial respiratory chain complexes I and IV lead to a substantial decline in ATP synthesis efficiency in ALS patients (Cheng et al., 2025). Mechanisms for clearing damaged organelles, such as mitochondrial autophagy, also become impaired. IGS2.7 is a small molecule capable of inhibiting mitophagy, which has been demonstrated to exert neuroprotective effects (Maestro et al., 2022). Abnormalities in various structural and functional aspects of mitochondria lead to insufficient cellular energy supply, excessive mitochondrial ROS production, and cascading amplification of oxidative stress and glutamate (Glu) excitotoxicity, thereby causing ALS-related pathological damage (Provenzano et al., 2023).

Based on the aforementioned pathological characteristics, mitochondrial dysfunction and oxidative stress form a self-reinforcing pathological loop. Dysfunctional mitochondria, particularly those with impaired electron transport chains, generate significantly higher levels of ROS than under normal conditions, directly exacerbating the cellular oxidative stress burden (Sever et al., 2022). On the other hand, ROS generated by ALS-related pathological factors such as cytoplasmic TDP-43 or FUS protein aggregates, or ROS from other pathways, in turn attack mitochondria and disrupt membrane contact communication between mitochondria and other organelles (such as the ER). This disrupts their physiological regulatory functions in autophagy, inflammation, energy metabolism, and ER stress (Chen et al., 2021; Jhanji et al., 2021).

### 3.3 Protein homeostasis imbalance

Proteins are essential components of cells and serve as the fundamental material basis for maintaining and regulating vital functions. Therefore, sustaining protein expression, folding, function, and localization in a stable and balanced state—that is, maintaining protein homeostasis—is crucial for ensuring the normal physiological activities of living organisms (Ciechanover and Kwon, 2017). Protein homeostasis is dynamically regulated primarily by two processes: protein synthesis and degradation. Protein synthesis serves as the initial stage in regulating protein homeostasis. Newly synthesized proteins undergo various post-translational modifications and are transported to different cellular locations, exhibiting functional diversity. Degradation, on the other hand, acts as a crucial terminal regulatory mechanism to prevent misfolded proteins or those with abnormal localization and accumulation. This process primarily involves molecular chaperones and the protein degradation system (Hipp et al., 2019). Molecular chaperones (such as the heat shock protein family) serve as the primary defense against protein misfolding and aggregation. Their main function is to assist newly synthesized polypeptide chains or misfolded proteins in achieving correct folding or refolding, thereby preventing non-specific aggregation. When misfolding cannot be corrected, molecular chaperones further guide these abnormal proteins into degradation pathways. Soluble misfolded proteins are typically targeted to the UPS for degradation, while insoluble large protein aggregates and damaged organelles are directed to the autophagy-lysosomal pathway for clearance (Ciechanover and Kwon, 2017; Dikic, 2017).

In ALS patients, this multi-tiered protein homeostasis control system experiences functional disruption at different stages. Under sustained stressors such as mutant protein production and oxidative stress, the Hsp system becomes overactivated or even depleted, diminishing its ability to repair misfolded proteins and losing its anti-aggregation function. This accelerates the formation of toxic aggregates like TDP-43 and SOD1. Arimoclomol, a co-inducer of the heat shock response, stabilizes the activated heat shock transcription factor 1 (HSF1) and upregulates the expression of several Hsp families. It has been reported to delay disease progression in preclinical ALS models and prevent the aggregation of mutant SOD1 (Kalmar and Greensmith, 2017).

The UPS system is frequently compromised when its core degradation pathway becomes obstructed by excess misfolded proteins originating upstream. This leads to the accumulation of highly toxic, undegraded ubiquitinated proteins and oligomers within the cytoplasm, disrupting multiple cellular functions such as RNA metabolism and transport. Consequently, this results in neuronal dysfunction and cell death (Scotter et al., 2014). Evidence indicates that loss of TDP-43 function associated with ALS can cause proteasome dysfunction, while inhibition of the proteasome in normal MNs can also induce cytoplasmic accumulation, aggregation, and insolubility of TDP-43, forming a vicious cycle (Van Eersel et al., 2011). Pharmacological activation of the UPS demonstrates the potential to clear TDP-43 and thereby attenuate its neurotoxicity (Tran and Lee, 2022). When UPS function is impaired or overwhelmed, cells increasingly rely on the autophagy-lysosomal pathway to clear larger protein aggregates and organelles. However, this backup system is also defective in ALS.

The autophagy–lysosomal pathway, as a key intracellular degradation mechanism, delivers macromolecular aggregates and damaged organelles to lysosomes for degradation through processes such as macroautophagy (i.e., the formation of autophagosomes encapsulating substrates). This system is crucial for maintaining neuronal homeostasis (Nikoletopoulou et al., 2015). In ALS, multifaceted dysfunction within this system directly drives disease pathology. First, initiation of the autophagy process and substrate recognition become impaired. ALS-associated gene mutations, such as those in tank-binding kinase 1 involved in autophagy initiation and optineurin (*OPTN*) and sequestosome 1 (*SQSTM1*, also known as *p62*), impair the efficient recognition and engulfment of toxic protein aggregates and damaged mitochondria by autophagosomes (Cirulli et al., 2015; Rudnick et al., 2017). Second, in MNs with extremely long axons, the transport and maturation of autophagosomes are highly susceptible to disruption. Autophagosomes generated by axonal autophagy rely on an intact retrograde transport system to reach the cell body and fuse with lysosomes. However, the axonal transport defects commonly observed in ALS lead to abnormal accumulation of autophagosomes within axons, hindering their subsequent degradation (Burk and Pasterkamp, 2019). Even if autophagosomes are successfully transported and fuse, their contents may fail to be ultimately digested due to functional defects such as insufficient acidification of the lysosome itself or inadequate enzyme activity (Root et al., 2021). It is evident that the macroautophagy pathway may experience comprehensive dysfunction across all stages, from initiation and transport to degradation. As a crucial selective form of autophagy, defects in mitochondrial autophagy play a particularly pivotal role in ALS pathogenesis. Mutations in *OPTN* and *p62/SQSTM1* have been demonstrated to directly impair this process, leading to the accumulation of dysfunctional mitochondria. This, in turn, generates ROS that exacerbate protein misfolding and overall cellular stress, creating a vicious cycle (Smith et al., 2019).

Ultimately, dysregulation across all levels of the protein homeostasis system leads to the accumulation of insoluble terminal inclusions composed of non-degradable toxic proteins such as TDP-43 and SOD1. These inclusions exert neurotoxic effects, disrupting neuronal homeostasis and ultimately causing the degeneration and death of MNs (Utami et al., 2025).

### 3.4 Endoplasmic reticulum stress

The ER is essential for protein synthesis, folding, modification, and calcium storage, and ER stress occurs when unfolded or misfolded proteins exceed the folding capacity of the molecular chaperone 78 kDa glucose-regulated protein, also known as binding immunoglobulin protein (GRP78/BiP) and the clearance capacity of ER-associated degradation system (Oakes and Papa, 2015). To restore ER homeostasis, cells initiate the unfolded protein response (UPR), a signaling network primarily dependent on three ER transmembrane sensors: inositol-requiring enzyme 1 alpha (IRE1α), protein kinase RNA-like endoplasmic reticulum kinase (PERK), and activating transcription factor 6 (ATF6). This response aims to suppress protein translation, upregulate molecular chaperones, and enhance the clearance of misfolded proteins (Mahdi et al., 2016).

Under physiological conditions, GRP78/BiP binds to IRE1α, PERK, and ATF6 and maintains them in an inactive state; upon accumulation of misfolded proteins, GRP78/BiP dissociates from these sensors and initiates UPR signaling (Lee, 2005). Dissociation frequently triggers the activity of the ribonuclease domain of IRE1, enabling the processed X-box binding protein 1 (XBP1) mRNAs to translocate into the nucleus. This promotes the transcriptional upregulation of adaptive UPR factors to degrade misfolded proteins (Junjappa et al., 2018; Riaz et al., 2020; Pincus et al., 2010; Yoshida et al., 2001). Upon activation following dissociation, PERK phosphorylates eukaryotic initiation factor 2α (eIF2α) at serine 51, generating phosphorylated eIF2α. This phosphorylation blocks the assembly of the pre-initiation complex on ribosomes, thus inhibiting global protein synthesis, while selectively promoting the expression of activating transcription factor 4 (ATF4) (Harding et al., 1999; Humeau et al., 2020). ATF4 is a transcription factor that promotes the expression of multiple genes involved in enhancing cellular recovery and adaptation to stress conditions. During the early stages or mild levels of ER stress, ATF4 synergizes with XBP1s produced by the IRE1α pathway to further induce a series of genes that contribute to alleviating ER stress (Rozpedek et al., 2016). ATF6 is transported into the Golgi apparatus, cleaved by proteases, and translocated to the nucleus, where it further induces the transcription of genes encoding ER chaperones and components of the ER-associated degradation pathway (Teske et al., 2011).

However, chronic or intense ER stress caused by the accumulation of large amounts of misfolded proteins shifts the UPR from a protective response to a pro-apoptotic signal, known as the “terminal UPR.” Beyond the long-term attenuation of overall protein synthesis disrupting cellular protein homeostasis, chronic stress activates the ATF4/C/EBP homologous protein (CHOP) axis. CHOP is a transcription factor that promotes cell death through multiple pathways, while sustained high expression of ATF4 drives cells toward apoptosis (Hu et al., 2019). In ALS fruit flies and mammalian cells, the neurotoxicity induced by TDP-43 overexpression was found to be directly associated with eIF2α phosphorylation and activation of the ATF4/CHOP pathway. Inhibiting this pathway using a PERK inhibitor (GSK2606414) effectively rescued neurodegeneration (Kim et al., 2014). Prolonged ER stress also causes crosstalk with mitochondria via the mitochondria-associated ER membrane (MAM), affecting mitochondrial phagocytosis and energy metabolism, ultimately leading to programmed cell death (Takeda et al., 2019).

ER stress in ALS is not restricted to motor neurons but also involves non-neuronal cells, including astrocytes and microglia, thereby contributing to non-cell-autonomous motor neuron injury (Sims et al., 2022). Mahadevan et al. discovered that activation of the UPR in astrocytes can induce UPR signaling in neighboring cancer cells, a phenomenon termed propagating ER stress (Mahadevan et al., 2012). Further studies revealed that treating astrocytes with ER stress inducers thapsigargin or tunicamycin significantly elevated the expression levels of GRP78, spliced XBP1, and CHOP in HT-22 hippocampal neurons via conditioned medium, confirming that astrocytes can transmit ER stress signals through soluble mediators. This intercellular transmission of UPR signaling was further validated in co-culture systems involving neurons, astrocytes, and microglia, demonstrating that ER stress can propagate throughout neural networks (Sprenkle et al., 2019). Additionally, astrocytes subjected to ER stress promote the expression of inflammatory cytokines such as interleukin-1β (IL-1β) and IL-6 in microglia through PERK-dependent pathways. They also reduce levels of protective markers, thereby driving microglia toward a pro-inflammatory phenotype (Meares et al., 2014; Guthrie et al., 2016). Specific knockout of *XBP1* in astrocytes has also been shown to reduce the number of infiltrating monocytes within the CNS and decrease IL-6 signaling, Nuclear factor kappa B (NF-κB) signaling, and the expression of chemokine-related genes in microglia (Wheeler et al., 2019). This further confirms that activation of the astrocytic UPR pathway can regulate the neuroinflammatory microenvironment. This phenotypic switch is closely associated with persistent ER stress and the resulting inflammatory signaling, forming a vicious feedback loop between neurons and glial cells. Therefore, restoring ER homeostasis, blocking the emergence of terminal UPR, and intervening in propagated ER stress signals are crucial for alleviating both neuronal autonomous and non-autonomous damage in ALS and delaying disease progression.

### 3.5 Neuroinflammation

Neuroinflammation and significant dysregulation of the central and peripheral immune systems play a pivotal role in the onset and progression of ALS. The persistent activation of resident immune cells within the CNS, excessive production of inflammatory cytokines, and infiltration of peripheral immune cells collectively shape a chronic pro-inflammatory microenvironment. This environment exacerbates motor neuron damage through multiple mechanisms, thereby driving disease progression. This pathological process evolves dynamically across disease stages, with the expression and function of different immune cells exhibiting significant spatiotemporal heterogeneity and dual roles (McCauley and Baloh, 2019; Calma et al., 2024).

#### 3.5.1 Resident glial cells of the CNS

Microglia constitute approximately 5%–12% of CNS cells and perform immune surveillance and defense functions within the CNS. Astrocytes, the most abundant glial cells in the CNS, regulate neurotransmitters, supply metabolites and energy, secrete neurotrophic factors, and participate in blood-brain barrier formation. As the primary resident immune cells in the CNS, microglia and astrocytes serve as core effectors in the neuroinflammatory response of ALS.

Under certain pathological conditions, microglia undergo rapid changes in morphology, transcriptional profiles, and phagocytic activity. They may acquire an anti-inflammatory phenotype to exert neuroprotective effects, or adopt a pro-inflammatory phenotype to exert neurotoxic effects (Masuda et al., 2019). Studies of *postmortem* samples from ALS patients and rodent models of ALS have revealed substantial evidence that microglia promote and sustain inflammation. In the spinal cords and CNS of ALS mice, expression of the glial activation markers cluster of differentiation 14 (CD14), CD18, and CD68 is increased (Henkel et al., 2004). Additionally, positron emission tomography scans have similarly detected activated microglia in the brains of ALS patients (Zürcher et al., 2015). The phenotype of microglia can change with disease progression. More microglia isolated from early-stage ALS mice exhibit a protective/anti-inflammatory (M2) phenotype, whereas more microglia isolated from end-stage disease display a toxic/pro-inflammatory (M1) phenotype (Clarke and Patani, 2020). This shift is closely associated with the excessive activation of the NF-κB signaling pathway, prompting microglia to massively release ROS and pro-inflammatory cytokines such as IL-1β, IL-6, and tumor necrosis factor α (TNF-α). These factors induce motor neuron apoptosis through mechanisms including the suppression of mitochondrial function. Specific knockout of NF-κB in microglia significantly prolongs the lifespan of ALS model mice, confirming its crucial role (Frakes et al., 2014). Activated microglia can also drive astrocytes to transition into a neurotoxic reactive state by secreting IL-1α, TNF-α, and complement component 1q (C1q), thereby forming a vicious inflammatory cycle (Liddelow et al., 2017). Knockout of IL-1α, TNF-α, and C1q in SOD1-G93A mice significantly reduces the generation of reactive astrocytes, thereby substantially prolonging the survival of diseased mice (Guttenplan et al., 2020).

Astrocytes also play a dual role in disease. In the early stages of disease, astrocytes activate NF-κB signaling to upregulate Wnt ligand expression, thereby inducing glial cell proliferation through paracrine mechanisms. This process confers neuroprotective effects, including enhanced phagocytic function (Ouali Alami et al., 2018). However, under sustained inflammatory stimulation, their phenotype shifts toward neurotoxic astrocytes resembling the A1 subtype. In the spinal cords of ALS patients and SOD1-G93A mice, expression of the A1 astrocyte marker complement component 3 is significantly upregulated (Liddelow et al., 2017). This may be related to the secretion of extracellular vesicles containing mutant proteins such as SOD1, p-TDP-43, and FUS, which can mediate neurotoxic effects (Upadhya et al., 2020). Astrocytes can also release soluble factors such as TNF-α, which directly promote MN apoptosis (Kia et al., 2018). The NOD-like receptor family pyrin domain-containing 3 (NLRP3) inflammasome is a cytoplasmic multiprotein complex composed of the innate immune receptor protein NLRP3, apoptosis-associated speck-like protein containing a CARD, and the inflammatory protease caspase-1. It induces gasdermin D-dependent pyroptosis and promotes the release of IL-1β and IL-18, contributing to innate immune defense and homeostasis maintenance. However, in the pathophysiology of ALS, neuroinflammation mediated by NLRP3 inflammasome activation originating from astrocytes plays a crucial role. Johann et al. detected elevated expression of NLRP3, IL-18, and active caspase-1 in both mSOD1 mouse spinal cords and *postmortem* tissues from ALS patients. They further demonstrated that NLRP3 inflammasomes and their associated components in ALS primarily originate from astrocytes within the spinal cord (Johann et al., 2015). These studies collectively indicate that activated astrocytes may accelerate ALS progression by enhancing inflammatory responses.

#### 3.5.2 Peripheral immune cells

The peripheral immune system, composed of innate and adaptive immune cells, also plays a crucial role in driving the onset and progression of ALS. Changes in their numbers, functional imbalances, and infiltration into the CNS profoundly influence the inflammatory microenvironment and the state of MNs in ALS.

Among innate immune cells, the role of monocytes/macrophages has garnered significant attention. Peripheral monocytes from ALS patients exhibit a pro-inflammatory state, demonstrating heightened susceptibility to activation and differentiation into an inflammatory phenotype. These cells produce elevated levels of IL-6 and TNF-α, with the concentration of these factors positively correlated with disease progression rate (Du et al., 2020). Notably, the function of these cells is context-dependent. In the early stages of disease, promoting monocyte infiltration into the CNS may exert neuroprotective effects. Using human immunoglobulin G or the CD95-Fc fusion protein to facilitate monocyte infiltration into the CNS increases the survival of MNs and delays disease onset (Zondler et al., 2016).

Regarding adaptive immune cells, T lymphocytes play a crucial role in the inflammatory response of ALS. CD4^+^ T cells, particularly regulatory T cells (Treg cells), maintain immune homeostasis in the early stages of the disease by releasing anti-inflammatory factors such as IL-4, suppressing pro-inflammatory T helper 1 (Th1) cells, and promoting microglial states associated with anti-inflammatory functions (Thonhoff et al., 2018). However, during the rapid progression of the disease, the number and suppressive function of Treg cells decrease and their function becomes impaired, weakening their protective role. Concurrently, the pro-inflammatory Th17-cell subset increases and levels of its characteristic cytokine IL-17A increase. The latter can directly impair motor neuron survival in a dose-dependent manner (Fu et al., 2022). The role of CD8^+^ T cells, however, appears contradictory. On one hand, CD8^+^ T cells infiltrating the CNS can indeed directly mediate apoptosis of MNs through cytotoxic pathways such as Fas- and granzyme-dependent cytotoxic pathways, exerting a negative impact on disease progression (Coque et al., 2019). On the other hand, in ALS mice, CD8^+^ T cells can be activated by damaged peripheral motor neuron axons and the major histocompatibility complex at the NMJ. This activation accelerates the clearance of damaged axonal debris, promotes myelin regeneration along axons and at the NMJ, delays muscle denervation, and prolongs survival (Liu et al., 2020). This indicates that the function of CD8^+^ T cells is highly dependent on the specific pathological microenvironment and cannot be simply characterized as purely harmful (Figure 3).

**FIGURE 3:**
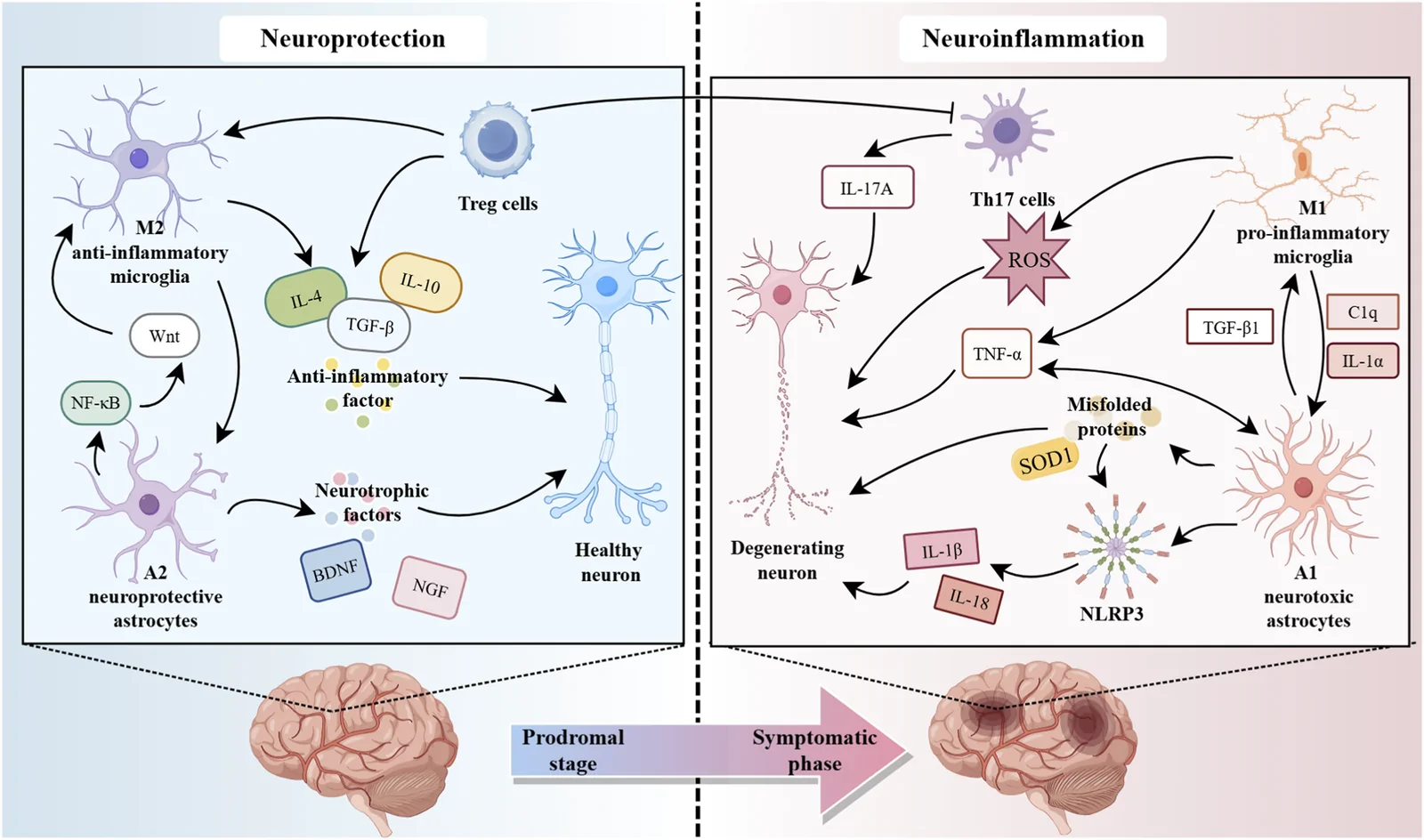
Neuroprotection and neuroinflammation in ALS. The left side illustrates anti-inflammatory and neuroprotective responses, whereas the right side shows neuroinflammatory and neurotoxic processes. Microglia, astrocytes, and infiltrating peripheral immune cells may exert context-dependent protective or detrimental effects during ALS progression. Shifts between M1-like pro-inflammatory and M2-like anti-inflammatory-associated microglial states reflect dynamic phenotypic changes rather than fixed cell identities. Th17, T helper cells 17; Treg, Regulatory T cells; IL-4, Interleukin-4; IL-10, Interleukin-10; TGF-β, Transforming growth factor-β; BDNF, Brain-derived neurotrophic factor; NGF, Nerve growth factor; Wnt, Wingless/Integrated; IL-17A, Interleukin-17A; TNF-α, Tumor necrosis factor-α; TGF-β1, Transforming growth factor-β1; C1q, Complement component 1q; IL-1α, Interleukin-1α; IL-18, Interleukin-18; SOD1, Superoxide dismutase 1; IL-1β, Interleukin-1β; NLRP3, NOD-like receptor family pyrin domain-containing 3. This figure was created with Figdraw (ID: SRPOS74047).

### 3.6 Glutamate excitotoxicity

Glutamate is the primary excitatory neurotransmitter in the CNS, playing a crucial role in maintaining neuronal baseline excitability, synaptic transmission, and higher cognitive functions (Pasanta et al., 2023). Under physiological conditions, glutamate is released primarily from presynaptic glutamatergic terminals and activates ionotropic and metabotropic receptors to mediate excitatory neurotransmission. Extracellular glutamate is rapidly cleared, predominantly through astrocytic excitatory amino acid transporters, thereby limiting excessive receptor activation and maintaining synaptic homeostasis (Neves et al., 2023).

However, in ALS, dysregulation of Glu signaling leads to its accumulation in the synaptic cleft, causing excessive activation of postsynaptic receptors. This triggers a cascade of events known as excitotoxicity, ultimately resulting in selective motor neuron death. The development of excitotoxicity is closely associated with increased presynaptic Glu release, reduced glial capacity for Glu reuptake, and alterations in the composition and function of postsynaptic receptors (Arnold et al., 2024).

In the SOD1-G93A mouse model, dysfunction of glutamatergic presynaptic terminals in the spinal cord has been demonstrated as a key driver of excitotoxicity. Bonifacino et al. found that metabotropic glutamate receptors 1/5 (mGluR1/5) are overexpressed during the symptomatic phase of early disease, leading to abnormal Ca^2+^ release from the ER and subsequently inducing excessive vesicular Glu release (Bonifacino et al., 2019). Furthermore, this chronic presynaptic calcium homeostasis imbalance may further trigger downstream signaling alterations such as the phosphorylation of synaptic protein I, promoting the mobilization of vesicles from the reserve pool to the readily releasable pool, thereby expanding the readily releasable Glu vesicle pool (Milanese et al., 2011). This hyperactive presynaptic release mechanism, combined with impaired synaptic Glu clearance due to reduced astrocytic Glu transporter function, collectively elevates synaptic Glu concentration. This excessively activates postsynaptic receptors, creating a persistent excitotoxic vicious cycle. Targeting this pathway, the drug riluzole has been demonstrated to reduce pathological neurotransmitter release by inhibiting voltage-gated Na^+^/Ca^2+^ channels, thereby partially delaying disease progression (Bellingham, 2011; Hollingworth et al., 2024).

Dysfunction of the astrocytic Glu transporter excitatory amino acid transporter 2 (EAAT2) is a key factor contributing to synaptic Glu accumulation. In sALS, approximately 60%–70% of patients exhibit significantly reduced EAAT2 protein expression in the motor cortex and spinal cord, partly attributed to abnormal splicing and decreased stability of EAAT2 mRNA (Lin et al., 1998). In fALS, mutant *SOD1* suppresses EAAT2 activity through oxidative damage, while *C9orf72* mutations result in its downregulation (Trotti et al., 1999; Howland et al., 2002; Fomin et al., 2018). Impaired EAAT2 function prolongs Glu reuptake in the synaptic cleft, leading to sustained activation of postsynaptic receptors, exacerbating calcium influx and neuronal damage. Although ceftriaxone increased EAAT2-related glutamate transport in preclinical studies, the Phase III trial failed to demonstrate benefit on the primary clinical outcomes, illustrating the difficulty of translating target engagement in glutamate handling into meaningful disease modification (Cudkowicz et al., 2014).

Intracellular Ca^2+^ overload-mediated signaling disruption in postsynaptic neurons is central to the execution of excitotoxicity. The α-amino-3-hydroxy-5-methyl-4-isoxazolepropionic acid receptor (AMPAR) is an ionotropic Glu receptor expressed on postsynaptic neurons. Glu in the synaptic cleft promotes Ca^2+^ influx into postsynaptic cells by activating and modulating AMPARs. Under physiological conditions, calcium-impermeable AMPARs typically remain impermeable to Ca^2+^ due to the presence of RNA-edited GluA2 subunits. However, in spinal cord tissue from ALS patients, reduced adenosine deaminase acting on RNA 2 (ADAR2) enzyme function or abnormal localization leads to insufficient GluA2 editing. This converts AMPARs to calcium-permeable forms, significantly increasing Ca^2+^ influx (Hosaka et al., 2021). MNs themselves have weak calcium buffering capacity and primarily rely on organelles such as mitochondria and the ER for calcium regulation. Excessive Ca^2+^ influx leads to mitochondrial calcium overload by activating the calcium-dependent protease calpain. This protease cleaves the TDP-43 protein into aggregation-prone fragments and degrades the mitochondrial outer membrane fusion protein mitofusin 2 (MFN2). Consequently, mitochondrial fragmentation and functional loss occur, inducing mitochondrial morphological abnormalities, respiratory chain dysfunction, and excessive ROS production (Yamashita and Kwak, 2014; Wang et al., 2015; 2019). ROS not only directly damages neuronal lipids, proteins, and DNA, but also diffuses to surrounding astrocytes, inhibiting EAAT2 function and further reducing Glu clearance, thereby forming a positive feedback loop (Rao et al., 2003). ER, serving as a crucial intracellular calcium reservoir, is deeply involved in excitotoxicity processes. Imbalances in Ca^2+^ homeostasis can trigger ER stress, activating UPR. ER stress and mitochondrial dysfunction exhibit close interaction, with calcium signaling exchange occurring via the MAM. Dysregulation of this pathway further exacerbates cellular calcium disruption and oxidative stress (Smith et al., 2019).

Glu excitotoxicity in ALS represents a multi-step, multi-cellular cascade process. Future interventions targeting ADAR2/GluA2 editing, EAAT2 expression regulation, calmodulin upregulation, or calpain inhibition may offer novel therapeutic opportunities for ALS.

### 3.7 Neuromuscular junction disorders and axonal transport defects

The NMJ is formed by the presynaptic motor neuron, postsynaptic muscle cell, and terminal Schwann cells, and converts motor-neuron action potentials into muscle contraction. Structural and functional abnormalities of the NMJ, including denervation and endplate fragmentation, are early pathological features of ALS (Chand et al., 2018; Bjornskov et al., 1984). In recent years, molecular biology research has revealed multiple molecular-level alterations at the NMJ, such as increased expression of axonal growth inhibitory factors and abnormal expression of acetylcholine receptor subunits (Bruneteau et al., 2015; Palma et al., 2016). The integrity of the axonal transport system is crucial for maintaining presynaptic function at the NMJ. Even molecular-level intervention targeting a single axonal transport regulatory protein can significantly alter axonal growth density and impair NMJ formation efficiency (Salzinger et al., 2025).

Axonal transport facilitates bidirectional movement of proteins, organelles, and signaling molecules between the cell body and distal synapses, forming the foundation for maintaining neuronal polarity and synaptic function. This process relies on the precise coordination of microtubule tracks and motor proteins such as kinesin, which mediates anterograde transport, and dynein, which mediates retrograde transport. Therefore, mutations in genes encoding these motor proteins can directly disrupt transport homeostasis, induce NMJ denervation, and constitute one of the pathogenic factors in ALS. As a core component of the critical retrograde transport motor complex, functional loss or mutation of the *DCTN1* gene disrupts axonal transport homeostasis. The underlying mechanisms span from classical retrograde transport defects and abnormal vesicle accumulation to newly identified synaptic-associated splicing disorders, ultimately converging to cause NMJ dysfunction and induce ALS-like pathology (Laird et al., 2008; Borg et al., 2023). Similarly, mutations in *KIF5A*, which encodes kinesin family member 5A, have been associated with an earlier age of onset in ALS. This may be related to alterations in the cargo-binding domain of the mutant protein, leading to abnormalities in axonal transport (Nicolas et al., 2018; Baron et al., 2022).

Additionally, abnormal aggregation of misfolded proteins such as mutant SOD1 and TDP-43 can directly or indirectly disrupt the axonal transport system. Mutant SOD1 protein activates p38 mitogen-activated protein kinase (MAPK), which phosphorylates kinesin and inhibits anterograde axonal transport (Morfini et al., 2013). Abnormal aggregation of TDP-43 not only upregulates histone deacetylase 6 expression, indirectly reducing microtubulin acetylation levels and impairing transport processes, but also disrupts retrograde transport of signal endosomes in axons by affecting dynein complex function or abnormally binding to motor proteins, thereby interfering with intracellular delivery of neurotrophic factors (Lee et al., 2020; Sleigh et al., 2020; Pulya et al., 2021). In ALS, brain-derived neurotrophic factor (BDNF) serves as a crucial reverse neurotrophic signal. Its transport from skeletal muscle to motor neuron somata via axons is essential for activating somatic tropomyosin receptor kinase B (TrkB) receptors and downstream survival pathways. Impairment of this transport process directly results in insufficient TrkB receptor activation and weakened neuroprotective signaling (Lanuza et al., 2019). Research confirms that the pathological environment of ALS can impair this signaling pathway. CSF from sporadic ALS patients can downregulate BDNF expression in NSC-34 cells *in vitro* and induce neurodegeneration, while exogenous supplementation of BDNF can mitigate this damage (Vijayalakshmi et al., 2009; Shruthi et al., 2017). *In vivo* studies further revealed that intrathecal injection of CSF from ALS patients significantly reduced *BDNF* mRNA levels in the spinal cord of newborn rats, while compensatory increases were observed in skeletal muscle expression (Deepa et al., 2011; Shanmukha et al., 2018).

In addition to the disruption of neurotrophic signaling, synaptic transmission at the neuromuscular junction itself and the homeostasis of postsynaptic skeletal muscle cells are also directly impaired. Unc-13 homolog A (UNC13A) is a crucial presynaptic protein that participates in maintaining synaptic active zones by enabling the initiation and docking of synaptic vesicles (Willemse et al., 2023). Research has revealed that loss of nuclear TDP-43 function, including in many C9orf72-associated ALS cases, promotes cryptic exon inclusion in UNC13A transcripts, thereby reducing functional UNC13A expression, impairing presynaptic vesicle release, disrupting synaptic transmission, and accelerating motor neuron degeneration (Brown et al., 2022; Fu et al., 2023). Pathological protein aggregates can also cause skeletal muscle mitochondrial dysfunction, ROS accumulation, and activation of inflammatory and apoptotic pathways, directly inducing denervation injury and muscle atrophy (Shanmukha et al., 2018).

Given the central role of axonal transport defects and NMJ dysfunction in the early stages of ALS, therapeutic strategies targeting this pathway have become a research hotspot (Ionescu et al., 2019). Current research suggests that axonal transport defects, disruption of neurotrophic signaling, and impaired synaptic transmission collectively form a self-reinforcing pathological loop that promotes NMJ degeneration. Future studies should further elucidate the interactions and spatiotemporal sequence among these mechanisms to identify more effective intervention targets.

Taken together, the pathological processes discussed above form an interconnected and self-reinforcing network rather than acting as isolated events. Reciprocal interactions among cellular stress responses, together with non-cell-autonomous and excitotoxic influences, may progressively amplify motor neuron vulnerability. These disturbances can subsequently compromise axonal function and NMJ integrity, allowing pathological consequences to propagate across the motor neuron–axon–NMJ–muscle unit and ultimately contribute to denervation, muscle weakness, and atrophy (Figure 4).

**FIGURE 4:**
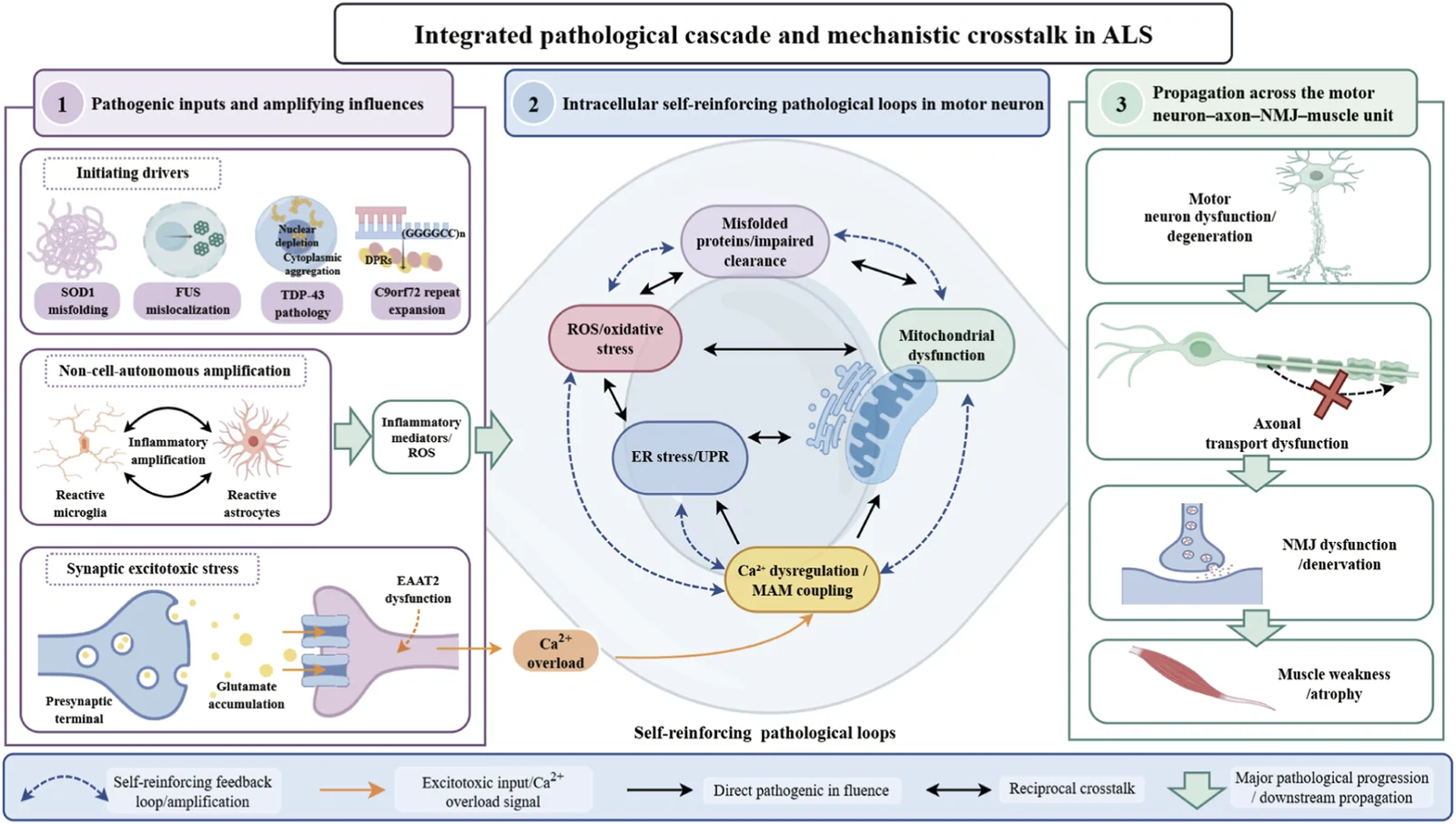
Integrated pathological cascade and mechanistic crosstalk in amyotrophic lateral sclerosis. ALS pathogenesis is conceptualized as a self-reinforcing network in which genetic/proteinopathic abnormalities, non-cell-autonomous inflammatory signals, and excitotoxic stress converge on motor-neuron homeostasis. Reciprocal interactions among impaired proteostasis, oxidative stress, mitochondrial dysfunction, ER stress, and Ca^2+^ dysregulation amplify cellular injury and progressively compromise the motor neuron–axon–NMJ–muscle unit, ultimately contributing to denervation, weakness, and muscle atrophy. Arrows indicate direct pathogenic effects, reciprocal crosstalk, feedback amplification, excitotoxic/Ca^2+^ inputs, and major downstream progression. ALS, Amyotrophic lateral sclerosis; SOD1, Superoxide dismutase 1; TDP-43, TAR DNA-binding protein 43; FUS, Fused in sarcoma; C9orf72, Chromosome 9 open reading frame 72; DPRs, Dipeptide repeat proteins; ROS, Reactive oxygen species; ER, Endoplasmic reticulum; UPR, Unfolded protein response; MAM, Mitochondria-associated ER membrane; Ca^2+^, Calcium ion; EAAT2, Excitatory amino acid transporter 2; NMJ, Neuromuscular junction. This figure was created with Figdraw (ID: YOITO42bb6).

## 4 Mechanism-oriented pharmacological evidence for TCBM interventions and plant-derived bioactive compounds in ALS

To provide a mechanism-oriented synthesis, TCBM interventions and selected plant-derived bioactive compounds are organized according to the major ALS-related pathological processes they were reported to modulate. Their molecular pathways, experimental models, principal outcomes, and translational evidence are summarized in Table 1, while botanical composition, extract type, dose/concentration, lowest reported effective dose or concentration, and treatment duration are provided in Table 2, following the same mechanism-oriented sequence as Table 1. Evidence for multi-pathway activity should be interpreted cautiously, as it may reflect either simultaneous effects within the same experimental system or convergence across independent studies; the latter should not be considered equivalent to experimentally demonstrated multi-target action.

### 4.1 Targeting glial activation and neuroinflammation

Neuroinflammation is a major pathological component of ALS and is primarily characterized by sustained activation of microglia and astrocytes and the excessive production of inflammatory mediators. Although glial responses may exert protective functions during the early stages of disease, persistent activation can establish a chronic pro-inflammatory microenvironment and amplify non-cell-autonomous motor neuron injury.

#### 4.1.1 Suppression of glial activation

Within this inflammatory cascade, astrocytic and microglial reactivity provide a cellular link between inflammatory signaling and motor neuron vulnerability. Reactive astrocytes may impair trophic and metabolic support to motor neurons, whereas activated microglia can sustain local inflammation through the continued release of cytokines and other neurotoxic mediators (Stacchiotti et al., 2025).

Wen-Pi-Tang, a traditional herbal formula composed of Aqueous extract of *Rheum officinale* Baill. [Polygonaceae; Rhei Rhizoma], *Panax ginseng* C.A.Mey. [Araliaceae; Ginseng Radix], *Aconitum japonicum* Thunb. [Ranunculaceae; Aconiti Tuber], *Zingiber officinale* Roscoe [Zingiberaceae; Zingiberis Rhizoma], and *Glycyrrhiza glabra* L. [Fabaceae; Glycyrrhizae Radix], attenuated glial activation in SOD1-G93A mice. Oral treatment dose-dependently reduced glial fibrillary acidic protein (GFAP)-positive astrocytes and CD11b-positive microglia in the lumbar spinal cord, particularly at 200 mg/kg, together with reduced motor neuron loss and delayed disease onset. The parallel reduction in microglial heme oxygenase-1 (HO-1) and inducible nitric oxide synthase (iNOS) further supports suppression of reactive gliosis as a potential contributor to its neuroprotective effects (Sekiya et al., 2009). Huolingshengji Formula is a compound herbal preparation containing *Astragalus mongholicus* Bunge [Fabaceae; Astragali Radix], Epimedii Herba (source taxon not specified in the ALS study), *Rehmannia glutinosa* (Gaertn.) DC. [Orobanchaceae; Rehmanniae Radix], *Cornus officinalis* Siebold & Zucc. [Cornaceae; Corni Fructus], *Atractylodes macrocephala* Koidz. [Asteraceae], and Poria (sclerotium; source taxon not specified). In male hSOD1-G93A mice, HLSJ treatment reduced microglial and astrocytic activation in the lumbar spinal cord, together with lower expression of the inflammation-related proteins TNF-α, cyclooxygenase-2, and iNOS. This attenuation of reactive gliosis coincided with greater motor neuron preservation and improved disease outcomes, supporting glial modulation as a potential contributor to the neuroprotective effects of HLSJ in ALS (Zhou et al., 2018).

Diallyl trisulfide is a garlic-oil organosulfur compound associated with *Allium sativum* L. [Amaryllidaceae]. In SOD1-G93A mice, diallyl trisulfide exerted neuroprotective effects. Oral diallyl trisulfide treatment initiated at disease onset enhanced HO-1 expression in the lumbar spinal cord and was accompanied by reduced GFAP expression, preservation of motor neurons, and improved motor performance. The concurrent induction of HO-1-associated stress responses and attenuation of reactive astrogliosis may underlie, at least in part, the neuroprotective effects of diallyl trisulfide in ALS (Guo et al., 2011).

#### 4.1.2 Inhibition of pro-inflammatory signaling and mediator production

Beyond glial activation, sustained neuroinflammation in ALS involves persistent innate immune signaling and inflammatory mediator production. Toll-like receptor 2 (TLR2) and Toll-like receptor 4 (TLR4) are upregulated in reactive glia, and TLR4 deletion prolongs survival in SOD1-G93A mice, supporting the involvement of TLR-associated signaling in disease progression (Casula et al., 2011; Lee et al., 2015). NF-κB and p38 MAPK signaling are likewise implicated in microglia-mediated motor neuron toxicity and glial responses (Tortarolo et al., 2003), while microglia-derived inflammatory mediators can further promote neurotoxic astrocyte reactivity (Guttenplan et al., 2020). Collectively, TLR-, NF-κB-, and MAPK-associated pathways emerge as recurrent inflammatory targets across the interventions reviewed below.

Gamisoyo-San is frequently employed to address symptoms like depression, night sweats, hot flashes, and palpitations, which overlap significantly with autonomic dysfunction that occurs in the later stages of ALS (Chen et al., 2022). In female hSOD1-G93A mice, Gamisoyo-San suppressed CD11b and TLR4 expression in the gastrocnemius muscle, accompanied by attenuation of local inflammation and neuromuscular pathological damage (Park and Yang, 2018). The anti-inflammatory activity of Jaeumganghwa-Tang in hSOD1-G93A mice was most evident in the spinal cord, where downregulation of ionized calcium-binding adaptor molecule 1, TLR4, and TNF-α suggested concurrent attenuation of microglial reactivity and TLR4-associated inflammatory signaling. The parallel improvement in molecular and functional outcomes suggests that attenuation of spinal neuroinflammation may contribute to its motor benefits (Lee and Yang, 2019).

Genistein is a plant-derived isoflavone occurring in soybean, *Glycine* max (L.) Merr. [Fabaceae], and other legumes (Du et al., 2018; Ahn et al., 2019). Genistein is orally absorbed but undergoes extensive phase II metabolism, particularly glucuronidation (Serebrenik et al., 2023). In SOD1-G93A mice, genistein attenuated spinal microgliosis and astrogliosis, accompanied by reduced TLR2, TLR4, NF-κB p65, and cyclooxygenase-2 expression and lower levels of the pro-inflammatory cytokines IL-1β, IL-6, and TNF-α. These anti-inflammatory changes coincided with greater spinal motor neuron survival, delayed disease onset, and improved motor performance (Zhao et al., 2019). Oxymatrine, a quinolizidine alkaloid extracted from the root of *Sophora flavescens* Aiton [Fabaceae], exerted anti-inflammatory and neuroprotective effects in female SOD1-G93A mice (Lin et al., 2022). Oxymatrine treatment attenuated spinal microgliosis and astrogliosis and reduced the expression of NF-κB, TNF-α, iNOS, and IL-1β, while increasing the anti-inflammatory markers CD206, Arg1, and IL-4. These changes were accompanied by greater lumbar motor neuron survival, delayed motor deterioration, and prolonged survival, supporting modulation of the pro-/anti-inflammatory balance as a potential contributor to Oxymatrine-mediated neuroprotection in ALS (Zhang et al., 2021).

#### 4.1.3 Modulation of microglial phenotype-associated responses

Microglial responses in ALS are heterogeneous and vary with anatomical region, disease progression, and genetic background. Recent single-cell and single-nucleus transcriptomic studies have identified multiple transcriptionally distinct microglial states in the ALS motor cortex and spinal cord, including populations associated with disease progression and genetic background (Masrori et al., 2025; Tuddenham et al., 2025). This heterogeneity suggests that neuroinflammation in ALS is driven by dynamic and context-dependent microglial states rather than a simple binary M1/M2 transition.

Miyako Bidens pilosa is a medicinal botanical preparation derived from a plant of the Asteraceae family and has been reported to attenuate spinal microglial and astrocytic activation and motor neuron loss in symptomatic SOD1-G93A mice (Kosuge et al., 2020). A subsequent study specifically examined microglia/macrophage phenotype-associated responses and found that miyako *Bidens pilosa* selectively reduced the M1-associated marker interferon-γ receptor and the pro-inflammatory cytokines TNF-α, IL-1β, and IL-6, while leaving the M2-associated marker largely unaffected. Miyako *B. pilosa* also suppressed lipopolysaccharide-induced pro-inflammatory cytokine expression in BV-2 microglia, supporting selective restraint of M1-like pro-inflammatory responses rather than generalized suppression of microglial activity (Tsuruta et al., 2023). Allicin is a garlic-derived organosulfur compound associated with *A. sativum* L. [Amaryllidaceae] (Cai S. et al., 2019). Allicin is chemically unstable and undergoes rapid transformation after ingestion, making systemic exposure strongly dependent on formulation and delivery strategy (Deng et al., 2023). In SOD1-G93A mice, allicin treatment reduced the abundance of CD86-positive M1-like microglia and increased Arg1-positive M2-like microglia. It also increased IL-10 and transforming growth factor beta (TGF-β) expression while decreasing IL-1β and TNF-α levels. These findings indicate attenuation of microglia-mediated neuroinflammation and a shift toward a less pro-inflammatory microglial state. The inflammatory changes were associated with a longer disease duration and reduced motor neuron loss in the anterior horn of the lumbar spinal cord (Gao et al., 2026). Promoting a less pro-inflammatory and more anti-inflammatory-associated microglial response represents another potential strategy for attenuating neuroinflammation.

### 4.2 Targeting oxidative stress and impaired antioxidant defense

Oxidative stress in ALS is characterized by both excessive production of reactive oxygen and nitrogen species and insufficient antioxidant and repair capacity. Accordingly, the interventions reviewed below are organized according to these processes.

#### 4.2.1 Suppression of oxidative-stress-generating systems

In addition to mitochondrial electron leakage, oxidative and nitrosative stress in ALS may be amplified by enzymatic sources such as nicotinamide adenine dinucleotide phosphate (NADPH) oxidase and inducible nitric oxide synthase.

KCHO-1, a 30% ethanol extract of nine traditionally used herbs, suppressed microglia-associated oxidative-stress-generating systems in hSOD1-G93A mice. Oral treatment reduced spinal ROS, iNOS, and gp91phox, a catalytic component of NADPH oxidase. Similar reductions in ROS, nitrite, iNOS, and gp91phox were observed in lipopolysaccharide/interferon-γ-activated BV-2 microglia, supporting inhibition of nitric oxide- and NADPH oxidase-derived oxidants as a potential mechanism (Kook et al., 2017).

Epigallocatechin-3-gallate, a major green-tea polyphenol from *Camellia sinensis* (L.) Kuntze [Theaceae], likewise reduced spinal iNOS and NF-κB expression and microglial activation in SOD1-G93A mice, accompanied by greater motor neuron preservation, delayed disease onset, and prolonged survival (Xu et al., 2006). These effects support suppression of iNOS-associated nitric oxide production as one potential component of epigallocatechin-3-gallate-mediated neuroprotection in ALS. A separate study further showed that epigallocatechin-3-gallate enhanced PI3K/Akt-associated survival signaling and reduced cytochrome c release, caspase-3 activation, and poly(ADP-ribose) polymerase cleavage, suggesting additional effects on downstream cell-survival and mitochondrial apoptotic pathways (Koh et al., 2006).

#### 4.2.2 Attenuation of lipid peroxidation and radical-mediated oxidative damage

Once generated, excessive ROS and reactive nitrogen species can propagate neuronal injury through free-radical reactions and membrane lipid peroxidation. Lipid peroxidation generates reactive aldehydes such as malondialdehyde (MDA) and 4-hydroxynonenal (4-HNE), which can further damage proteins and membrane components; thus, reducing these products provides a relatively direct indicator of attenuation of oxidative injury.

Rosmarinic acid is a plant-derived phenolic acid occurring in multiple Lamiaceae species, and *Perilla frutescens* (L.) Britton [Lamiaceae] is one reported medicinal source. It possesses antioxidant and anti-inflammatory properties but is extensively metabolized after oral intake; clinically relevant CNS exposure remains uncertain (Deguchi and Ito, 2020; Hitl et al., 2021). An earlier study showed that low-dose intraperitoneal rosmarinic acid improved motor performance, attenuated motor neuron degeneration, and prolonged survival in SOD1-G93A mice (Shimojo et al., 2010). A subsequent study provided more direct evidence of antioxidant activity: oral rosmarinic acid significantly reduced spinal 4-HNE, while preserving choline acetyltransferase (ChAT)-positive motor neurons and delaying neurological deterioration (Seo et al., 2015). SK-PC-B70M is an oleanolic-glycoside saponin fraction isolated from the root of *Pulsatilla cernua* var. *koreana* (Y.Yabe ex Nakai) Y.N.Lee [Ranunculaceae], similarly reduced lipid-peroxidative damage in SOD1-G93A mice. Treatment significantly decreased spinal MDA and 4-HNE accumulation and preserved ventral-horn neurons, together with improvements in motor function. Its protective effect against H_2_O_2_-induced injury in SH-SY5Y cells further supports an antioxidant component of its neuroprotective activity (Seo et al., 2011). EGb 761 is a standardized leaf extract of Ginkgo biloba L. [Ginkgoaceae] and has been reported to possess nitric-oxide-scavenging and neuroprotective properties (Yu et al., 2015). Oral administration of 0.022% or 0.045% EGb 761 through the diet was reported to improve motor performance and preserved anterior-horn motor neurons in SOD1-G93A mice, with more evident effects in males. However, its proposed antioxidant mechanism remains indirect because oxidative biomarkers were not directly assessed in this ALS model (Ferrante et al., 2001).

#### 4.2.3 Enhancement of endogenous antioxidant defenses

ALS-related oxidative injury is also associated with inadequate endogenous defenses, including reduced Nrf2 activity, disturbed glutathione metabolism, and insufficient antioxidant-enzyme capacity.

Shenqi Fuzheng Injection is an injectable botanical preparation composed of *Codonopsis pilosula* (Franch.) Nannf. [Campanulaceae; Codonopsis Radix] and Astragali Radix, for which *A. mongholicus* Bunge [Fabaceae] is the pharmacopoeial source, at a crude-drug ratio of 1:1. In SOD1-G93A mice, Shenqi Fuzheng Injection activated Nrf2-associated signaling, increased downstream antioxidant enzymes, and reduced MDA and ROS-related damage in SOD1-G93A mice. These molecular changes were accompanied by reduced motor neuron loss and prolonged survival (Sugimoto et al., 2021). Antioxidant-enzyme changes were reported in both the CNS and peripheral blood, providing concordant evidence across compartments, although the relationship between these biochemical changes and CNS exposure remains unresolved.

γ-Oryzanol is a mixture of ferulic-acid steryl and triterpenyl esters associated with rice bran and rice bran oil from Oryza sativa L. [Poaceae]. In hSOD1-G85R *Drosophila* and hSOD1-G93A-expressing NSC-34 cells, γ-oryzanol enhanced antioxidant capacity and attenuated mutant-SOD1-associated neurotoxicity (Abate et al., 2024). These effects were associated with Akt activation and upregulation of Nrf2/glutamate-cysteine ligase catalytic subunit and STAT3/Hsp70, suggesting that γ-oryzanol may alleviate oxidative injury by promoting glutathione synthesis and strengthening endogenous enzymatic antioxidant defenses (Zhang et al., 2019a). Fisetin is a plant-derived flavonoid occurring in multiple botanical sources. Its oral bioavailability is limited by low solubility and rapid metabolism, and clinically attainable CNS exposure remains uncertain (Ding et al., 2022; Krishnakumar et al., 2022; Szymczak and Cielecka-Piontek, 2023). In mutant hSOD1 models, fisetin activated ERK-associated signaling, increased catalase, glutathione peroxidase, and HO-1, and reduced ROS-related injury, supporting enhancement of endogenous antioxidant responses (Wang et al., 2018). Apigenin is a plant-derived flavonoid, which shows therapeutic potential in various neurodegenerative diseases due to its antioxidant and anti-inflammatory properties (Waheed et al., 2023). Studies have demonstrated that apigenin upregulates ALDH1A2 and activates Nrf2/ARE signaling while limiting oxidative-stress-related apoptosis, effects that may contribute to spinal motor neuron preservation (Liang et al., 2024).

### 4.3 Targeting mitochondrial dysfunction and bioenergetic failure

Motor neurons have high energy demands, and the maintenance of their physiological functions depends heavily on mitochondrial dynamics and bioenergetic capacity. In ALS, mitochondrial abnormalities include structural disruption, reduced activities of respiratory chain complexes I and IV, impaired ATP synthesis, and defective clearance of damaged organelles. These changes can further increase mitochondrial ROS production and disrupt calcium homeostasis, thereby reinforcing oxidative stress, excitotoxicity, and motor neuron degeneration.

#### 4.3.1 Modulation of mitochondrial biogenesis and fusion-associated homeostasis

Peroxisome proliferator-activated receptor gamma coactivator-1 alpha (PGC-1α)-mediated mitochondrial biogenesis and MFN2/OPA1-dependent fusion are essential for maintaining mitochondrial abundance, network integrity, and respiratory capacity. Their disruption may promote bioenergetic failure and accumulation of dysfunctional mitochondria, making restoration of mitochondrial biogenesis and fusion-associated homeostasis a potential therapeutic strategy.

Honokiol, a biphenolic compound isolated from the roots, stems, or bark of *Magnolia officinalis* Rehder & E.H. Wilson [Magnoliaceae], has antioxidant and anti-inflammatory properties (Li et al., 2024). In SOD1-G93A-expressing NSC-34 cells, honokiol increased the expression of the mitochondrial biogenesis-related proteins PGC-1α, NRF1, and TFAM and restored the fusion-related proteins OPA1 and MFN2, while improving mitochondrial membrane potential, ATP production, respiratory-chain complex II and IV activities, and mitochondrial morphology. In male SOD1-G93A mice, it similarly increased spinal PGC-1α, NRF1, TFAM, and OPA1 expression, accompanied by improved motor function and prolonged survival (Zhou et al., 2023). These findings support coordinated enhancement of mitochondrial biogenesis, fusion, and bioenergetic capacity.

Resveratrol, a polyphenol found in multiple botanical sources, such as Japanese knotweed, *Reynoutria japonica* Houtt. [Polygonaceae; syn. *Polygonum cuspidatum* Siebold & Zucc.], appears to regulate mitochondrial homeostasis through an integrated sirtuin 1 (SIRT1)–AMP-activated protein kinase (AMPK)-dependent metabolic response rather than antioxidant activity alone (Chi et al., 2012). In mutant hSOD1-G93A motor neuron-like cells, resveratrol restored SIRT1 signaling, increased ATP, and upregulated PGC-1α and MFN2; attenuation of these effects by SIRT1 inhibition supports a mechanistic link between SIRT1 activation and mitochondrial recovery (Wang et al., 2011). In SOD1-G93A mice, resveratrol increased spinal SIRT1 and AMPK activity, normalized respiratory-chain protein expression, and modulated Fis1, LC3-II, and Beclin-1, suggesting coordinated regulation of mitochondrial adaptation and autophagy-associated organelle homeostasis, together with delayed disease onset and prolonged survival (Mancuso et al., 2014). Restoration of SIRT1/AMPK activity and NAD levels in ALS patient-derived mesenchymal stem cells further supports engagement of this energy-sensing network, although direct mitochondrial function was not assessed (Yun et al., 2018). Resveratrol also enhanced SIRT1-mediated HSF1 deacetylation and Hsp25/Hsp70 induction, suggesting convergence between mitochondrial homeostasis and proteostatic defense (Han et al., 2012). However, lower-dose dietary resveratrol failed to improve motor performance or survival, indicating that efficacy is sensitive to dose, administration route, exposure, and bioavailability (Markert et al., 2010). Overall, the available evidence supports effects of resveratrol on mitochondrial bioenergetics, biogenesis, and quality-control-associated pathways, although direct assessment of mitochondrial respiration and mitochondria-specific autophagic flux remain limited.

Tetramethylpyrazine nitrone (TBN) is a synthetic nitrone derivative of tetramethylpyrazine. Tetramethylpyrazine itself is associated with *Ligusticum chuanxiong Hort. [*Apiaceae*; Chuanxiong Rhizoma]*. TBN has demonstrated antioxidant and neuroprotective effects in preclinical studies and was retained as a notable example of translation from ALS-specific preclinical evidence to clinical evaluation (Bahbah, 2026). Oral TBN is rapidly absorbed, has a short half-life without appreciable accumulation, and shows reduced systemic exposure with food; preclinical studies also support blood–brain barrier penetration (Zhang et al., 2018; Zhu et al., 2024). In SOD1-G93A mice, TBN activated the PGC-1α/Nrf2/HO-1 pathway, reduced ROS and MDA, attenuated glial activation and motor neuron loss, and improved motor dysfunction (Wen et al., 2021). In a TDP-43M337V mouse model, it improved motor deficits and early cognitive impairment and prolonged survival, accompanied by modulation of Akt/mTOR/GSK-3β and AMPK/PGC-1α/Nrf2 signaling and reduced TDP-43 expression (Huang et al., 2021). These findings suggest coordinated regulation of mitochondrial biogenesis, metabolic signaling, and antioxidant defense.

#### 4.3.2 Modulation of mitochondrial morphology and organelle homeostasis

Mitochondrial morphology is closely linked to organelle quality control and cellular energy homeostasis. In ALS, mitochondrial swelling, cristae disruption, and accumulation of damaged organelles may reflect impaired mitochondrial maintenance. Because structural improvement does not necessarily indicate restored bioenergetic function or mitophagic flux, morphological findings should ideally be supported by functional measures such as respiratory-chain activity, membrane potential, ATP production, calcium handling, or organelle turnover.

Wen-Shen-Jian-Pi prescription is a study-reported seven-drug formula containing Ginseng Radix (15 g), Astragali Radix (15 g), Cistanches Herba (30 g), Atractylodis Macrocephalae Rhizoma (15 g), Poria (15 g), Glycyrrhizae Radix (10 g), and Epimedii Herba (25 g). The primary ALS study did not report unique authenticated source taxa for all seven botanical drugs. Wen-Shen-Jian-Pi prescription was evaluated in conditional Adar2 knockout mice, a mechanistic model of sporadic ALS characterized by deficient ADAR2-mediated GluA2 Q/R-site editing and Ca^2+^-permeable AMPA receptor-associated motor neuron degeneration. Twelve weeks of oral treatment slowed weight loss, improved limb mobility and muscle strength, and reduced motor neuron death associated with mitochondrial and autophagic pathology. Ultrastructural analysis showed more morphologically normal mitochondria and less autophagosome accumulation, supporting preservation of mitochondrial morphology and organelle homeostasis (Gong et al., 2022).

Kaempferol, a dietary flavonol found in several plants including *Crocus sativus* L. [Iridaceae], has limited oral exposure because of extensive first-pass glucuronidation. After a dietary dose of approximately 9 mg, peak plasma concentrations reached only about 0.1 μM and consisted mainly of kaempferol-3-glucuronide (DuPont et al., 2004; Wang R. et al., 2024). In C9orf72-associated ALS models, kaempferol enhanced ER–mitochondrial coupling through GRP75-mediated IP3R–VDAC1 signaling, restored mitochondrial calcium uptake, and increased respiratory-chain complex I and IV activities and ATP5J expression (Pilotto et al., 2025). These findings indicate that targeting ER–mitochondrial communication and calcium handling may help alleviate bioenergetic dysfunction in C9orf72-associated ALS.

### 4.4 Targeting impaired proteostasis and endoplasmic reticulum stress

Protein homeostasis depends on coordinated protein synthesis, chaperone-assisted folding, and removal of damaged or misfolded proteins through the UPS and autophagy–lysosomal pathways. In ALS, disruption of these systems promotes accumulation of pathogenic proteins such as mutant SOD1, TDP-43, and FUS, while persistent protein misfolding can further disturb ER homeostasis and trigger maladaptive stress responses (Kabashi et al., 2012; Zhao et al., 2022). Experimental evidence indicates that several TCBM interventions and plant-derived bioactive compounds may act at different levels of this proteostasis network.

#### 4.4.1 Promotion of UPS-mediated clearance of misfolded proteins

The UPS is a major pathway for eliminating short-lived, damaged, and misfolded proteins, and impaired proteasomal activity may contribute to pathogenic protein accumulation in ALS. Enhancing UPS-dependent degradation may therefore reduce intracellular protein burden and help restore proteostatic balance.

Lycorine is an Amaryllidaceae alkaloid reported from the bulbs of Lycoris radiata (L’Hér.) Herb. [Amaryllidaceae] (Xiao et al., 2022). In TDP-43A315T models, lycorine activated cyclic adenosine monophosphate/protein kinase A (cAMP/PKA) signaling and promoted UPS-mediated degradation of pathogenic TDP-43, while suppressing new protein synthesis, thereby reducing TDP-43A315T levels and improving TDP-43-associated neuropathology and motor-related phenotypes (Wen et al., 2024). These findings provide relatively direct evidence that coordinated enhancement of protein degradation and reduction of protein synthesis can lower pathogenic TDP-43 burden.

Baicalein, a flavonoid derived from the root of *Scutellaria baicalensis* Georgi [Lamiaceae], reduced soluble and insoluble mutant SOD1 and intracellular aggregation in SOD1-G93A-transfected NSC-34 cells. It increased K48-linked ubiquitinated proteins without marked changes in p62 or LC3B. Baicalein also reduced extracellular release of misfolded SOD1. In SOD1-G93A mice, these effects were accompanied by lower intraneuronal mSOD1 burden, greater spinal motor neuron preservation, and partial preservation of NMJ innervation and motor performance (Ting et al., 2025). This pattern further supports preferential involvement of proteasome-associated rather than autophagic clearance.

#### 4.4.2 Modulation of autophagy–lysosomal pathways and aggregate clearance

The autophagy–lysosomal system complements the UPS by removing protein aggregates and damaged cellular components. Several botanical interventions alter LC3, p62/SQSTM1, mTOR, and related markers in ALS models; however, changes in these static markers alone do not establish enhanced autophagic flux, which requires dynamic assessment of autophagosome formation and degradation.

The Achyranthes bidentata, Gastrodia elata, and Chaenomeles sinensis formulation, a botanical extract composed of *Achyranthes bidentata* Blume [Amaranthaceae], *Gastrodia elata* Blume [Orchidaceae], and *Pseudocydonia sinensis* (Dum.Cours.) C.K.Schneid. [Rosaceae; reported as *Chaenomeles sinensis* in the primary ALS study], reduced p62, LC3B, TGF-β, SMAD2, HO-1, ferritin, and Bax in male hSOD1-G93A mice, together with improvements in motor and muscle pathology (Yang, 2025). The changes in p62 and LC3B are consistent with modulation of autophagy-associated proteostasis, whereas the accompanying TGF-β/SMAD2, oxidative-stress, and apoptosis-related changes suggest broader effects on muscle and neuronal pathology.

An aqueous extract of *P. sinensis* (Dum.Cours.) C.K.Schneid. [Rosaceae], reported as *C. sinensis* in the primary ALS study, similarly reduced p62/SQSTM1, LC3B, phosphorylated mTOR, and SMAD2 in hSOD1-G93A mice, together with lower TLR4, IL-1β, HO-1, and Bax and improvement of muscle dysfunction and motor decline (Yang and Lee, 2023). An aqueous extract of Paeonia lactiflora Pall. [Paeoniaceae] was also evaluated in hSOD1-G93A mice (Wu et al., 2010). Treatment reduced the expression of proteins associated with inflammation and oxidative stress in skeletal muscle and spinal cord, and regulated autophagy function by reducing the levels of SQSTM1, microtubule-associated protein 1A/1B light chain 3B, and SMAD family member 2. These changes were associated with attenuation of SOD1 aggregate-related neurotoxicity (Yang and Lee, 2025). Pu-erh tea extract is a fermented tea extract. The extract provides more direct evidence for lysosome-dependent clearance of ALS-associated proteins. In cells expressing R521C FUS/TLS, the extract interacted with FET family proteins, including FUS/TLS, EWS, and TAF15, and promoted their degradation without altering FUS/TLS mRNA. Degradation was attenuated by the lysosomal inhibitor ammonium chloride but not by the proteasome inhibitor lactacystin, supporting a lysosome-dependent mechanism. Treatment also reduced cytoplasmic accumulation of mutant FUS/TLS and its incorporation into stress granules under stress conditions (Yu et al., 2014). The inhibitor experiment provides stronger mechanistic support for lysosomal clearance than studies based solely on LC3 or p62 changes, although autophagic flux itself was not directly quantified.

Berberine is an isoquinoline alkaloid which was isolated from the rhizome of the Chinese herb *Coptis chinensis* Franch. [Ranunculaceae] (Song et al., 2020). It has very low oral bioavailability (<1%) because of poor solubility and permeability, P-glycoprotein-mediated efflux, and extensive first-pass metabolism; although detected in rat brain, clinically relevant human CNS exposure remains undefined (Tan et al., 2013; Cui et al., 2024). In a cellular model of TDP-43 proteinopathy, berberine modulated mTOR/p70S6K signaling, increased autophagy-associated degradation, and reduced insoluble TDP-43, supporting engagement of autophagic clearance pathways (Chang et al., 2016).

#### 4.4.3 Enhancement of chaperone responses and attenuation of ER stress

Molecular chaperones assist protein folding and limit aggregation, whereas persistent misfolded-protein accumulation can overwhelm ER folding capacity and activate the UPR. When unresolved, prolonged ER stress may shift from adaptive signaling toward cellular dysfunction and death. Enhancing chaperone capacity and limiting maladaptive ER stress therefore represent complementary proteostasis strategies.

Celastrol, a triterpenoid from the root bark of *Tripterygium wilfordii* Hook.f. [Celastraceae], has relatively low oral bioavailability in rats (∼17%) but can cross the blood–brain barrier, while human pharmacokinetic and CNS exposure data remain unavailable (Zhang et al., 2012; Shandra et al., 2025). In SOD1-G93A mice, celastrol prolonged HSF1 activation and increased Hsp70, providing direct evidence of enhanced heat-shock responses. Treatment also reduced TNF-α, iNOS, CD40, and GFAP and was associated with reduced motor neuron injury and improved disease-related outcomes (Kiaei et al., 2005). Although these findings indicate broader anti-inflammatory actions, HSF1 activation and Hsp70 induction provide the clearest link to chaperone-mediated proteostasis. In addition to its effects on mitochondrial homeostasis, kaempferol showed direct activity against ER stress in C9orf72-associated ALS models, suppressing tunicamycin-induced BiP elevation and ER-stress-associated neuronal death in patient-derived motor neurons and reducing phosphorylated eIF2α-associated UPR signaling in symptomatic C9-500 mice (Pilotto et al., 2025).

### 4.5 Targeting glutamate excitotoxicity

Glutamate excitotoxicity in ALS is driven largely by impaired glutamate clearance and excessive receptor-mediated calcium influx, which increase motor neuron vulnerability. However, direct pharmacological evidence that the interventions reviewed here modulate glutamate handling in ALS-specific models remains limited.

Nordihydroguaiaretic acid (NDGA) is a plant-derived lignan abundant in Larrea tridentata (Sessé & Moc. Ex DC.) Coville [Zygophyllaceae]. Among the interventions reviewed here, NDGA provides some of the most direct evidence for pharmacological modulation of glutamate uptake (Arteaga et al., 2005). In MN-1 neural cells, nordihydroguaiaretic acid dose-dependently enhanced high-affinity glutamate uptake, with 4 μM increasing the EAAT2-sensitive component by approximately threefold; pharmacological experiments further implicated 5-lipoxygenase-associated regulation. In SOD1-G93A mice treated from disease onset, nordihydroguaiaretic acid initially increased spinal glutamate uptake by approximately 50% after 10 days, but this effect was no longer evident after 20–30 days and did not translate into prolonged survival. The loss of sustained target engagement coincided with progressive upregulation of spinal P-glycoprotein, suggesting that disease-associated drug efflux may contribute to reduced CNS exposure and therapeutic efficacy (Boston-Howes et al., 2008). However, the transient nature of enhanced glutamate uptake in SOD1-G93A mice and the absence of a survival benefit highlight the substantial translational gap between short-term target engagement and meaningful disease modification.

### 4.6 Targeting NMJ injury, muscle denervation, and impaired neurotrophic support

Disruption of motor neuron–muscle connectivity is an early and functionally important feature of ALS. Progressive NMJ denervation, impaired neurotrophic support, and skeletal muscle degeneration can precede extensive motor neuron loss and contribute to progressive weakness. Available studies of the interventions reviewed here have mainly focused on preserving NMJ integrity and muscle structure or enhancing neurotrophic signaling, whereas direct evidence for restoration of axonal transport remains limited.

#### 4.6.1 Preservation of NMJ integrity and muscle–motor neuron connectivity

NMJ denervation progressively disrupts communication between motor axons and skeletal muscle, making preservation of neuromuscular connectivity a relevant therapeutic target.

Bojungikgi-Tang, a traditional multibotanical formula, attenuated muscle wasting and NMJ denervation in hSOD1-G93A mice (Shih et al., 2000; Li et al., 2022). Treatment preserved gastrocnemius morphology, reduced TGF-β and Prox1, increased α-bungarotoxin-positive NMJ structures by approximately 2.8-fold compared with untreated transgenic mice, and partially normalized abnormal β-catenin and phosphorylated GSK-3β (Ser9) expression in skeletal muscle, suggesting preservation of neuromuscular integrity through Wnt-related signaling (Cai M. et al., 2019). A four-botanical extract containing *Glycyrrhiza uralensis* Fisch. Ex DC. [Fabaceae], *A. macrocephala* Koidz. [Asteraceae], *P. ginseng* C.A.Mey. [Araliaceae], and *A. mongholicus* Bunge [Fabaceae], similarly increased tibialis anterior and gastrocnemius muscle weight and increased NMJ structures by approximately 2.4-fold. It also reduced inflammatory, oxidative-stress, and apoptosis-related markers, preserved spinal ChAT-positive motor neurons, and improved motor performance and stride length (Lee et al., 2021). Thus, its effects extended from skeletal muscle and NMJs to spinal motor neuron preservation.

Protocatechuic acid, a plant-derived phenolic acid and anthocyanin metabolite occurring in multiple botanical sources, also preserved gastrocnemius muscle weight and NMJ integrity in hSOD1-G93A mice, as shown by improved α-bungarotoxin/VAChT overlap and NMJ morphology. These changes were accompanied by greater lumbar α-motor neuron survival and improved motor performance (Hu et al., 2020; Koza et al., 2020). Human studies indicate that orally administered protocatechuic acid is systemically available, with free compound reaching approximately 3.27 μM in serum, while *in vitro* data support blood–brain barrier permeability (Zheng et al., 2019; Gallardo-Fernandez et al., 2024).

#### 4.6.2 Enhancement of neurotrophic signaling and motor neuron support

Impaired neurotrophic support may increase motor neuron vulnerability in ALS by compromising neuronal survival, synaptic maintenance, and functional connectivity. Enhancing neurotrophic signaling pathways, including BDNF/TrkB- and LPA receptor-associated mechanisms, may therefore provide an upstream strategy to support motor neuron integrity and synaptic function.

7,8-Dihydroxyflavone, a flavonoid isolated from *Oroxylum indicum (L.) Kurz [*Bignoniaceae*]*, activates TrkB and downstream signaling, thereby mimicking key aspects of BDNF activity. In SOD1-G93A mice, this mechanism was associated with enhanced neuronal differentiation and survival, increased dendritic spine density and synaptic connectivity, and improved motor phenotypes (Korkmaz et al., 2014; Nandhakumar et al., 2020). Gintonin is a gintonin-enriched glycolipoprotein fraction derived from *P. ginseng* C.A.Mey. [Araliaceae] and acts as an exogenous ligand of lysophosphatidic acid receptors (Nah, 2012). Preclinical studies support intestinal absorption and enhanced blood–brain barrier permeability, although quantitative human pharmacokinetics and clinically attainable CNS exposure remain undefined (Kim et al., 2018; Lee et al., 2018). In SOD1-G93A mice, long-term oral gintonin restored spinal LPA1 receptor expression and increased BDNF- and ChAT-immunoreactive cells, together with preservation of Nissl- and NeuN-positive neurons. These effects were accompanied by delayed motor dysfunction and prolonged survival (Nam et al., 2021). These findings suggest that LPA1-related regulation, together with enhanced neurotrophic and cholinergic support, may contribute to spinal motor neuron preservation in ALS.

## 5 Clinical evidence and current translational status

Although the preclinical evidence summarized above supports the modulation of several ALS-related pathological processes by TCBM interventions and selected plant-derived bioactive compounds, clinical evidence remains limited. Earlier studies were generally small and exploratory, whereas several recent investigations have adopted more rigorous randomized and multicenter designs. Accordingly, the available human evidence should be interpreted primarily as an indication of clinical feasibility and potential therapeutic activity rather than as confirmation of disease-modifying efficacy.

An early randomized study of Jiawei Sijunzi decoction in 48 patients reported a possible functional benefit in patients with limb-onset ALS, but interpretation was limited by the small sample size and open-label design (Pan et al., 2013). More recently, HLSJ was evaluated in a multicenter, randomized, double-blind phase II trial involving 140 patients. Although the primary Amyotrophic Lateral Sclerosis Functional Rating Scale-Revised (ALSFRS-R) endpoint did not demonstrate statistically significant superiority over riluzole, exploratory analyses suggested a slower functional decline, supporting further investigation rather than established clinical efficacy (Liu X. et al., 2026). Curcumin, a polyphenolic compound derived primarily from the rhizome of *Curcuma longa* L. (turmeric), was evaluated in a randomized, double-blind study involving 42 patients with ALS. Oral curcumin (600 mg/day) was associated with relative stabilization of ALSFRS-R scores and reduced oxidative stress markers, although the small sample size and short follow-up warrant cautious interpretation (Chico et al., 2018).

TBN has also entered clinical evaluation. In a phase II randomized placebo-controlled trial involving 155 patients, neither the low-dose TBN (600 mg twice daily) nor the high-dose TBN (1,200 mg twice daily) improved the primary ALSFRS-R endpoint compared with placebo. However, high-dose TBN showed exploratory benefits in grip strength and selected subgroup outcomes and was generally well tolerated. These secondary findings should therefore be regarded as hypothesis-generating rather than evidence of established clinical efficacy (Liu et al., 2025).

Systematic reviews and meta-analyses likewise suggest possible short-term functional benefits of Chinese herbal medicine in ALS, but the overall certainty of evidence remains limited. A meta-analysis including 20 studies and 1,218 participants rated much of the available evidence as low or very low certainty because of risk of bias and imprecision (Song et al., 2022). Another analysis of 14 randomized trials involving 1,141 participants reported improvements in some ALSFRS-related outcomes, but no clear benefit for mortality or quality of life, with substantial limitations in randomization, blinding, follow-up duration, and intervention heterogeneity (Liao et al., 2022).

## 6 Discussion: translational challenges and future directions

TCBM interventions and selected plant-derived bioactive compounds show recurrent effects on neuroinflammation, antioxidant defense, mitochondrial/metabolic homeostasis, and proteostasis. However, most evidence remains preclinical, and such cross-study convergence does not establish a shared causal mechanism. Null or dose-dependent findings, together with negative primary outcomes in recent HLSJ and TBN trials, indicate that the evidence is not uniformly positive. Publication bias cannot be excluded, and these recurrent signals should therefore be regarded as hypothesis-generating rather than established therapeutic mechanisms.

Beyond these limitations of the available literature, the present review also has a methodological limitation related to language restriction. The structured evidence set was restricted to English-language publications to maintain consistency in screening and data extraction across the international databases. Nevertheless, this approach may have excluded relevant Chinese-language pharmacological and clinical studies and introduced language-selection bias. Therefore, the current synthesis should be interpreted as representing the English-language, internationally indexed evidence base. Future systematic reviews incorporating major Chinese-language databases and bilingual screening may help determine whether inclusion of these studies materially alters the strength or clinical interpretation of the evidence.

At the level of the underlying preclinical evidence, another major limitation is the dependence on a relatively narrow range of experimental systems, particularly SOD1-G93A models. This problem extends beyond the interventions reviewed here: numerous agents that showed apparently beneficial effects in mutant-SOD1 mice subsequently failed to demonstrate comparable efficacy in human ALS, highlighting limitations in model validity, experimental design, and pharmacological translation (McGoldrick et al., 2013; De Cock et al., 2024). ALS is clinically and molecularly heterogeneous, and most cases are sporadic rather than attributable to SOD1 mutations. Although TDP-43-, C9orf72-, FUS-, and ADAR2-related systems broaden the mechanistic spectrum, these models reproduce distinct and only partially overlapping features of human ALS. SOD1 models primarily capture mutant-protein toxicity, oxidative stress, mitochondrial dysfunction, and motor-neuron degeneration, whereas TDP-43 and FUS models better reflect RNA-processing, protein-aggregation, and related cellular stress abnormalities. C9orf72 models uniquely reproduce repeat-RNA and dipeptide-repeat toxicity, while ADAR2-deficient models provide a mechanistic representation of sporadic ALS through impaired GluA2 editing and Ca^2+^-permeable AMPA receptor-mediated degeneration. Therefore, findings replicated across mechanistically distinct models provide stronger translational support than effects observed in a single genetic system. Similarly, immortalized motor-neuron-like and glial cell lines are useful for pathway dissection but cannot reproduce the multicellular interactions or patient-specific molecular backgrounds of ALS. Greater use of patient-derived induced pluripotent stem cell models and multicellular systems incorporating motor neurons and glial cells may improve disease relevance, particularly when combined with validation across mechanistically distinct *in vivo* models (Sances et al., 2016; Vahsen et al., 2022).

A second challenge concerns the distinction between administered dose and pharmacologically relevant exposure. Several compounds reviewed here are affected by poor oral bioavailability, extensive first-pass metabolism, rapid clearance, transporter-mediated efflux, or incompletely characterized blood–brain barrier penetration. Consequently, an effective dose in an animal model does not necessarily imply that comparable pharmacologically active exposure can be achieved in humans. Human-equivalent-dose conversion based on body weight or body-surface-area scaling is useful for initial dose estimation but cannot substitute for pharmacokinetic evaluation of interspecies differences in drug disposition and exposure (Blanchard and Smoliga, 2015). This issue is particularly important for CNS-directed interventions because systemic exposure alone does not establish adequate drug availability in the brain or spinal cord, and CSF concentrations provide only an imperfect surrogate for CNS exposure (Srinivas et al., 2018). Future studies should therefore move beyond dose–response relationships based solely on administered dose and, where feasible, relate systemic and CNS exposure to pharmacodynamic effects and functional outcomes, consistent with recommendations for ALS drug development (Berry et al., 2014). The feasibility of integrating plasma and CSF exposure with pharmacodynamic and target-engagement measures has been demonstrated in an early-phase ALS trial of apilimod (Babu et al., 2024).

Safety and pharmacodynamic monitoring represent additional priorities, particularly because some of the interventions reviewed here may ultimately be evaluated as adjuncts to established ALS therapies. Evidence regarding interactions with riluzole, edaravone, tofersen, and other disease-modifying treatments remains limited. Notably, in hSOD1-G93A mice, co-administration of Bojungikgi-Tang with riluzole did not produce overt hepatotoxicity or alter the measured hepatic drug-metabolism-related markers, while improving inflammatory and metabolic abnormalities (Park and Yang, 2022). Additional preclinical studies reported enhanced effects of this combination on muscle inflammation, autophagy, metabolic dysfunction, and TDP-43-associated pathology (Cai and Yang, 2022; Yang, 2024), suggesting potential pharmacodynamic complementarity. However, these studies did not establish formal pharmacokinetic interactions or clinical synergy. Future studies should therefore separately assess pharmacokinetic effects on drug disposition and systemic or CNS exposure and pharmacodynamic interactions producing additive, antagonistic, or safety-relevant effects under clinically relevant co-treatment conditions.

Future trials should also incorporate pharmacodynamic biomarkers to determine whether the proposed biological processes are modified in patients. Neurofilament light chain (NfL) is among the most established fluid biomarkers of neuroaxonal injury in ALS and has demonstrated treatment responsiveness, although it should not be considered a stand-alone surrogate for clinical efficacy (Miller et al., 2022; Benatar et al., 2023). GFAP may provide complementary information on astrocytic injury or reactivity, whereas CSF soluble triggering receptor expressed on myeloid cells 2 remains a candidate microglia-related marker because findings across ALS cohorts have been inconsistent (Jericó et al., 2023; Rosén et al., 2024). YKL-40 and C-reactive protein have shown associations with disease progression and treatment-related changes but remain relatively nonspecific inflammatory markers (Bowser et al., 2024). Thus, mechanism-related pharmacodynamic markers should ideally be interpreted together with neurodegeneration biomarkers and conventional clinical outcomes.

An additional challenge is the intrinsic complexity of multicomponent botanical preparations. Differences in botanical species, medicinal parts, cultivation conditions, extraction procedures, constituent proportions, and manufacturing processes may substantially influence chemical composition and pharmacological activity. As emphasized in current phytopharmacological reporting recommendations, future studies should provide appropriate botanical authentication, medicinal-part information, voucher documentation where applicable, extraction procedures, quantified marker constituents, chromatographic fingerprints, and evidence of batch-to-batch consistency (Heinrich et al., 2022). These considerations are directly relevant to regulatory development because manufacturing consistency and chemical reproducibility are prerequisites for meaningful comparison between preclinical preparations and clinical products. Regulatory development will further require predefined release specifications and stability criteria to ensure consistent identity, composition, and potency of the final product. Clinical batches should also demonstrate adequate batch-to-batch comparability throughout clinical development. Importantly, multicomponent composition should also not be equated with experimentally demonstrated synergy. For most ALS-related formulas reviewed here, synergistic or additive interactions among individual constituents have been inferred from the activity of the complete preparation rather than established through factorial combination studies, constituent-depletion/reconstitution experiments, isobolographic analysis, or related interaction methods. Similar methodological concerns have been identified more broadly in investigations of herbal-drug synergy (Zhou et al., 2016). Accordingly, future work should distinguish pharmacological complexity from experimentally validated synergism and should define the active constituents, exposure profiles, and reproducible chemical characteristics of candidate formulations before advanced clinical development.

Future studies should therefore prioritize mechanistic depth, model relevance, and translational validation rather than simply accumulating additional pathway-associated observations. Candidate interventions should, where feasible, be evaluated across complementary ALS models, including patient-derived induced pluripotent stem cell models and genetically or mechanistically distinct *in vivo* models. In addition, multicellular systems incorporating motor neurons and glial cells may better reflect the non-cell-autonomous mechanisms of ALS. Mechanistic claims should progress from pathway-associated observations toward causal validation using target perturbation, rescue, or pharmacological approaches. Functional assays should also be aligned with the proposed mechanisms rather than relying solely on molecular pathway changes. Clinical development should similarly proceed in stages, first establishing tolerability, clinically relevant exposure, CNS availability when required, and pharmacodynamic or target-engagement evidence before undertaking adequately powered efficacy trials. Recent ALS therapeutic trials have increasingly incorporated biomarker-guided strategies to determine biological activity before interpreting clinical outcomes (Miller et al., 2022). Prospective stratification according to genotype, disease stage, site of onset, or progression rate may help address ALS heterogeneity. ALSFRS-R decline remains an important functional endpoint, while respiratory outcomes, survival measures, and biomarkers such as NfL should be incorporated according to trial phase and duration (Van Den Berg et al., 2019).

Collectively, successful translation will require evidence that a well-characterized and reproducible candidate intervention acts in a disease-relevant experimental system, achieves pharmacologically meaningful exposure, engages the intended biological process, and ultimately produces clinically meaningful benefit.

## 7 Conclusion

Current evidence indicates that TCBM interventions and selected plant-derived bioactive compounds can modulate several ALS-related pathological processes in experimental systems, particularly neuroinflammation, oxidative stress, mitochondrial dysfunction, and impaired proteostasis. However, the evidence remains predominantly preclinical, is heavily dependent on a limited range of disease models, and does not establish clinical efficacy. Existing human studies provide preliminary evidence of feasibility and potential biological activity rather than confirmed disease-modifying benefit. Future translation will require reproducible botanical standardization, pharmacokinetic and CNS-exposure characterization, validation across disease-relevant models, biomarker-supported target engagement, and adequately powered clinical trials.

## Glossary

4-HNE: 4-hydroxynonenal
ADAR2: adenosine deaminase acting on RNA 2
ALS: amyotrophic lateral sclerosis
ALSFRS-R: Amyotrophic Lateral Sclerosis Functional Rating Scale-Revised
AMPK: AMP-activated protein kinase
AMPAR: α-amino-3-hydroxy-5-methyl-4-isoxazolepropionic acid receptor
ATF4: activating transcription factor 4
ATF6: activating transcription factor 6
ATP: adenosine triphosphate
Bax: Bcl-2-associated X protein
BDNF: brain-derived neurotrophic factor
C1q: complement component 1q
C9orf72: chromosome 9 open reading frame 72
cAMP: cyclic adenosine monophosphate
CD: cluster of differentiation
ChAT: choline acetyltransferase
CHOP: C/EBP homologous protein
CNS: central nervous system
CSF: cerebrospinal fluid
EAAT2: excitatory amino acid transporter 2
eIF2α: eukaryotic initiation factor 2 alpha
ER: endoplasmic reticulum
fALS: familial amyotrophic lateral sclerosis
FDA: U.S. Food and Drug Administration
FUS: fused in sarcoma
GFAP: glial fibrillary acidic protein
Glu: glutamate
GRP78/BiP: 78-kDa glucose-regulated protein/binding immunoglobulin protein
GSH: glutathione
HLSJ: Huolingshengji Formula
HO-1: heme oxygenase-1
HSF1: heat shock transcription factor 1
Hsp70: heat shock protein 70
IL: interleukin
iNOS: inducible nitric oxide synthase
IRE1α: inositol-requiring enzyme 1 alpha
MAM: mitochondria-associated endoplasmic reticulum membrane
MAPK: mitogen-activated protein kinase
MDA: malondialdehyde
MFN2: mitofusin 2
MNs: motor neurons
mRNA: messenger RNA
mTOR: mechanistic target of rapamycin
NADPH: nicotinamide adenine dinucleotide phosphate
NfL: neurofilament light chain
NF-κB: nuclear factor kappa B
NLRP3: NOD-like receptor family pyrin domain-containing 3
NMJ: neuromuscular junction
Nrf2: nuclear factor erythroid 2-related factor 2
OPTN: optineurin
PERK: protein kinase RNA-like endoplasmic reticulum kinase
PGC-1α: peroxisome proliferator-activated receptor gamma coactivator-1 alpha
PKA: protein kinase A
PRISMA: Preferred Reporting Items for Systematic Reviews and Meta-Analyses
ROS: reactive oxygen species
sALS: sporadic amyotrophic lateral sclerosis
SIRT1: sirtuin 1
SOD1: superoxide dismutase 1
SQSTM1: sequestosome 1
TARDBP: TAR DNA-binding protein gene
TBN: tetramethylpyrazine nitrone
TCBM: traditional Chinese botanical medicine
TCM: traditional Chinese medicine
TDP-43: TAR DNA-binding protein 43
TGF-β: transforming growth factor beta
TLR2: Toll-like receptor 2
TLR4: Toll-like receptor 4
TNF-α: tumor necrosis factor alpha
Treg: regulatory T cell
TrkB: tropomyosin receptor kinase B
UNC13A: Unc-13 homolog A
UPR: unfolded protein response
UPS: ubiquitin–proteasome system
XBP1: X-box binding protein 1
