# Supplementary material for: Traditional Chinese botanical medicines and plant-derived bioactive compounds in amyotrophic lateral sclerosis: mechanistic evidence, translational challenges, and therapeutic opportunities
Source: Front Pharmacol. 2026 Sep 24;17:1918967. doi: 10.3389/fphar.2026.1918967 (PMC13646943; doi:10.3389/fphar.2026.1918967)
Supplement: Supplementary file 1 [file Table1.pdf]

Supplementary Table S1. Database-specific literature search strategies

Searches were organized into broad intervention terms (A1), targeted candidate intervention names (A2), and ALS pathophysiology/mechanism searches (B). The publication-date cutoff used for PubMed and Web of Science was May 31, 2026. Record counts are those documented in the supplied search histories/exports before deduplication.

S1A. Search-yield summary

Database	Search sets	Records	Comment
**PubMed**	A1 = 427; A2 = 169; final B = 8,057	8,653	
**Web of Science Core Collection**	A1 = 458; A2 = 236; final B = 5,363	6,057	
**ScienceDirect**	A1 = 50; A2-1 = 12; A2-2 = 42; B1-B6 = 2,179	2,283	
**Total**		16,993	Before deduplication

S1B. PubMed: intervention-focused searches

Set	Exact search strategy	Results	Notes
**A1**	("Amyotrophic Lateral Sclerosis"[Mesh] OR "amyotrophic lateral sclerosis"[tiab]) AND ("Medicine, Chinese Traditional"[Mesh] OR "Phytotherapy"[Mesh] OR "Plants, Medicinal"[Mesh] OR "Plant Extracts"[Mesh] OR "Phytochemicals"[Mesh] OR "traditional Chinese medicine"[tiab] OR "Chinese herbal medicine"[tiab] OR "Chinese medicine"[tiab] OR "Chinese patent medicine"[tiab] OR "herbal medicine"[tiab] OR "herbal formula*"[tiab] OR "herbal preparation*"[tiab] OR "botanical medicine"[tiab] OR "medicinal botanical*"[tiab] OR "herbal extract*"[tiab] OR "plant extract*"[tiab] OR "natural product*"[tiab] OR "natural compound*"[tiab] OR phytochemical*[tiab] OR "plant-derived compound*"[tiab] OR "plant-derived bioactive compound*"[tiab] OR polyphenol*[tiab] OR flavonoid*[tiab] OR isoflavone*[tiab] OR alkaloid*[tiab] OR terpenoid*[tiab] OR "phenolic compound*"[tiab]) AND ("1900/01/01"[Date - Publication] : "2026/05/31"[Date - Publication])	427	Publication date: 1900/01/01-2026/05/31
**A2**	("Amyotrophic Lateral Sclerosis"[Mesh] OR "amyotrophic lateral sclerosis"[tiab]) AND ("Wen-Pi-Tang"[tiab] OR "Huolingshengji"[tiab] OR "diallyl trisulfide"[tiab] OR "Gamisoyo-San"[tiab] OR "Jaeumganghwa-Tang"[tiab] OR genistein[tiab] OR oxymatrine[tiab] OR "Bidens pilosa"[tiab] OR allicin[tiab] OR "KCHO-1"[tiab] OR "epigallocatechin-3-gallate"[tiab] OR EGCG[tiab] OR "rosmarinic acid"[tiab] OR "SK-PC-B70M"[tiab] OR EGb761[tiab] OR "Shenqi Fuzheng"[tiab] OR "oryzanol"[tiab] OR fisetin[tiab] OR apigenin[tiab] OR honokiol[tiab] OR resveratrol[tiab] OR "tetramethylpyrazine nitrone"[tiab] OR "Wen-Shen-Jian-Pi"[tiab] OR kaempferol[tiab] OR lycorine[tiab] OR baicalein[tiab] OR "Pseudocydonia sinensis"[tiab] OR "Paeonia lactiflora"[tiab] OR "Pu-erh tea"[tiab] OR berberine[tiab] OR celastrol[tiab] OR "nordihydroguaiaretic acid"[tiab] OR NDGA[tiab] OR "Bojungikgi-Tang"[tiab] OR "protocatechuic acid"[tiab] OR "7,8-dihydroxyflavone"[tiab] OR gintonin[tiab]) AND ("1900/01/01"[Date - Publication] : "2026/05/31"[Date - Publication])	169	Publication date: 1900/01/01-2026/05/31

S1C. PubMed: ALS pathophysiology/mechanism search (B)

Set	Exact search strategy	Results	Notes
**#1**	("Amyotrophic Lateral Sclerosis"[Mesh] OR "amyotrophic lateral sclerosis"[tiab])	41,302	
**#2**	("Disease Models, Animal"[Mesh] OR "Cells, Cultured"[Mesh] OR "Induced Pluripotent Stem Cells"[Mesh] OR "in vitro"[tiab]	5,230,477	

Set	Exact search strategy	Results	Notes
	OR "in vivo"[tiab] OR "ex vivo"[tiab] OR transgenic[tiab] OR knockout[tiab] OR knockdown[tiab] OR overexpress*[tiab] OR "animal model"[tiab] OR "animal models"[tiab] OR "mouse model"[tiab] OR "mouse models"[tiab] OR "murine model"[tiab] OR "murine models"[tiab] OR "rat model"[tiab] OR "rat models"[tiab] OR "cell model"[tiab] OR "cell models"[tiab] OR "cellular model"[tiab] OR "cellular models"[tiab] OR "cell culture"[tiab] OR "cell cultures"[tiab] OR "primary culture"[tiab] OR "primary cultures"[tiab] OR iPSC*[tiab] OR "induced pluripotent stem cell"[tiab] OR "induced pluripotent stem cells"[tiab] OR organoid*[tiab] OR "patient-derived"[tiab] OR "patient derived"[tiab] OR fibroblast*[tiab] OR "human tissue"[tiab] OR "human tissues"[tiab] OR "patient sample"[tiab] OR "patient samples"[tiab] OR postmortem[tiab] OR "post-mortem"[tiab] OR autops*[tiab] OR Drosophila[tiab] OR zebrafish[tiab])		
**#3**	((SOD1[tiab] OR TARDBP[tiab] OR "TDP-43"[tiab] OR FUS[tiab] OR C9orf72[tiab]) AND (mutat*[tiab] OR mutant*[tiab] OR variant*[tiab] OR "repeat expansion"[tiab] OR "repeat expansions"[tiab] OR "hexanucleotide repeat"[tiab] OR "hexanucleotide repeat expansion"[tiab] OR "gain of function"[tiab] OR "loss of function"[tiab])) OR "GGGGCC repeat"[tiab] OR "GGGGCC repeats"[tiab] OR "G4C2 repeat"[tiab] OR "G4C2 repeats"[tiab] OR "C9orf72 repeat expansion"[tiab] OR "C9orf72 repeat expansions"[tiab]	9,632	
**#4**	(proteinopath*[tiab] OR "protein misfolding"[tiab] OR "misfolded protein"[tiab] OR "misfolded proteins"[tiab] OR "protein aggregation"[tiab] OR "protein aggregates"[tiab] OR ((SOD1[tiab] OR TARDBP[tiab] OR "TDP-43"[tiab] OR FUS[tiab]) AND (misfold*[tiab] OR aggregat*[tiab] OR accumulation[tiab] OR inclusion*[tiab] OR oligomer*[tiab])) OR "stress granule"[tiab] OR "stress granules"[tiab] OR "RNA foci"[tiab] OR "dipeptide repeat"[tiab] OR "dipeptide repeats"[tiab])	40,599	
**#5**	#1 AND #2 AND (#3 OR #4)	5,422	
**#6**	(neuroinflamm*[tiab] OR ((microgli*[tiab] OR astrocyt*[tiab]) AND (activat*[tiab] OR reactiv*[tiab] OR inflamm*[tiab] OR cytokine*[tiab] OR phenotype*[tiab])))	131,681	
**#7**	("oxidative stress"[tiab] OR "reactive oxygen species"[tiab] OR redox[tiab] OR (mitochondri*[tiab] AND (dysfunction*[tiab] OR homeostas*[tiab] OR dynamic*[tiab] OR fission[tiab] OR fusion[tiab] OR bioenergetic*[tiab] OR respiration[tiab] OR "membrane potential"[tiab])))	721,492	
**#8**	(proteostas*[tiab] OR autophag*[tiab] OR "protein clearance"[tiab] OR "ubiquitin-proteasome"[tiab] OR "ubiquitin proteasome"[tiab] OR "proteasome dysfunction"[tiab] OR "endoplasmic reticulum stress"[tiab] OR "ER stress"[tiab] OR "unfolded protein response"[tiab])	164,352	
**#9**	(excitotox*[tiab] OR (glutamat*[tiab] AND (toxic*[tiab] OR transporter*[tiab] OR uptake[tiab] OR clearance[tiab] OR receptor*[tiab])) OR EAAT2[tiab] OR SLC1A2[tiab] OR "calcium dysregulation"[tiab])	88,611	
**#10**	("neuromuscular junction"[tiab] OR "neuromuscular junctions"[tiab] OR "motor endplate"[tiab] OR "motor endplates"[tiab] OR "motor end plate"[tiab] OR "motor end plates"[tiab] OR "axonal transport"[tiab] OR "axonal dysfunction"[tiab] OR axonopath*[tiab] OR denervation[tiab] OR "neuromuscular denervation"[tiab])	50,274	
**#11**	#1 AND #2 AND #6	1,655	

Set	Exact search strategy	Results	Notes
**#12**	#1 AND #2 AND #7	1,775	
**#13**	#1 AND #2 AND #8	1,130	
**#14**	#1 AND #2 AND #9	864	
**#15**	#1 AND #2 AND #10	1,016	
**#16**	#5 OR #11 OR #12 OR #13 OR #14 OR #15	8,268	Union of mechanism-domain searches
**#17**	(#16) AND english[lang] AND ("1900/01/01"[Date - Publication] : "2026/05/31"[Date - Publication])	8,057	Final B set; English; publication date through May 31, 2026

S1D. Web of Science Core Collection: intervention-focused searches

Set	Exact search strategy	Results	Notes
**A1**	TS=(<br>"amyotrophic lateral sclerosis"<br>)<br>AND<br>TS=(<br>"traditional Chinese medicine"<br>OR "Chinese herbal medicine"<br>OR "Chinese medicine"<br>OR "Chinese patent medicine"<br>OR "herbal medicine"<br>OR "herbal formula*"<br>OR "herbal preparation*"<br>OR "botanical medicine"<br>OR "medicinal botanical*"<br>OR "herbal extract*"<br>OR "plant extract*"<br>OR "natural product*"<br>OR "natural compound*"<br>OR phytochemical*<br>OR "plant-derived compound*"<br>OR "plant-derived bioactive compound*"<br>OR polyphenol*<br>OR flavonoid*<br>OR isoflavone*<br>OR alkaloid*<br>OR terpenoid*<br>OR "phenolic compound*"<br>)<br>AND DOP=1900-01-01/2026-05-31	458	Web of Science Core Collection
**A2**	TS=("amyotrophic lateral sclerosis")<br>AND<br>TS=(<br>"Wen-Pi-Tang"<br>OR Huolingshengji<br>OR "diallyl trisulfide"<br>OR "Gamisoyo-San"<br>OR "Jaeumganghwa-Tang"<br>OR genistein<br>OR oxymatrine<br>OR "Bidens pilosa"<br>OR allicin<br>OR "KCHO-1"<br>OR "epigallocatechin-3-gallate"<br>OR EGCG<br>OR "rosmarinic acid"<br>OR "SK-PC-B70M"<br>OR EGb761<br>OR "Shenqi Fuzheng"<br>OR oryzanol	236	Web of Science Core Collection

Set	Exact search strategy	Results	Notes
	OR fisetin<br>OR apigenin<br>OR honokiol<br>OR resveratrol<br>OR "tetramethylpyrazine nitrone"<br>OR "Wen-Shen-Jian-Pi"<br>OR kaempferol<br>OR lycorine<br>OR baicalein<br>OR "Pseudocydonia sinensis"<br>OR "Paeonia lactiflora"<br>OR "Pu-erh tea"<br>OR berberine<br>OR celastrol<br>OR "nordihydroguaiaretic acid"<br>OR NDGA<br>OR "Bojungikgi-Tang"<br>OR "protocatechuic acid"<br>OR "7,8-dihydroxyflavone"<br>OR gintonin<br>)<br>AND DOP=1900-01-01/2026-05-31		

S1E. Web of Science Core Collection: ALS pathophysiology/mechanism search (B)

Set	Exact search strategy	Results	Notes
**#1**	TI=("amyotrophic lateral sclerosis")<br>OR AB=("amyotrophic lateral sclerosis")	31865	
**#2**	AB=(<br>"in vitro"<br>OR "in vivo"<br>OR "ex vivo"<br>OR transgenic<br>OR mutant*<br>OR knockout<br>OR knockdown<br>OR overexpress*<br>OR "animal model*"<br>OR "mouse model*"<br>OR "murine model*"<br>OR "rat model*"<br>OR "cell model*"<br>OR "cellular model*"<br>OR "cell culture*"<br>OR "primary culture*"<br>OR iPSC*<br>OR "induced pluripotent stem cell*"<br>OR organoid*<br>OR "patient-derived"<br>OR "patient derived"<br>OR fibroblast*<br>OR "human tissue*"<br>OR "patient sample*"<br>OR postmortem<br>OR "post-mortem"<br>OR autops*<br>OR Drosophila<br>OR zebrafish<br>)	3153258	
**#3**	AB=(<br>proteinopath*<br>OR<br>(protein NEAR/3 (misfold* OR aggregat*))	48573	

Set	Exact search strategy	Results	Notes
	OR<br>(<br>(SOD1 OR "TDP-43" OR TARDBP OR FUS OR C9orf72)<br>NEAR/5<br>(misfold* OR aggregat* OR accumulation OR toxic* OR pathology)<br>)<br>OR "dipeptide repeat*"<br>OR "RNA foci"<br>OR "stress granule*"<br>)		
**#4**	AB=(<br>neuroinflamm*<br>OR<br>(<br>(microgli* OR astrocyt*)<br>NEAR/5<br>(activat* OR reactiv* OR inflamm* OR cytokine* OR phenotype*)<br>)<br>)	84727	
**#5**	AB=(<br>"oxidative stress"<br>OR "reactive oxygen species"<br>OR redox<br>OR<br>(<br>mitochondri*<br>NEAR/5<br>(<br>dysfunction*<br>OR homeostas*<br>OR dynamic*<br>OR fission<br>OR fusion<br>OR bioenergetic*<br>OR respiration<br>OR "membrane potential"<br>)<br>)<br>)	747207	
**#6**	AB=(<br>proteostas*<br>OR autophag*<br>OR "protein clearance"<br>OR "ubiquitin-proteasome"<br>OR "ubiquitin proteasome"<br>OR "proteasome dysfunction"<br>OR "endoplasmic reticulum stress"<br>OR "ER stress"<br>OR "unfolded protein response"<br>)	146042	
**#7**	AB=(<br>excitotox*<br>OR<br>(<br>glutamat*<br>NEAR/5<br>(toxic* OR transporter* OR uptake OR clearance OR receptor*)<br>)<br>OR EAAT2<br>OR SLC1A2<br>OR "calcium dysregulation"	43863	

Set	Exact search strategy	Results	Notes
	)		
**#8**	AB=(<br>"neuromuscular junction*"<br>OR "motor endplate*"<br>OR "motor end plate*"<br>OR "axonal transport"<br>OR "axonal dysfunction"<br>OR axonopath*<br>OR denervation<br>OR "neuromuscular denervation"<br>)	22139	
**#9**	#1 AND #2 AND #3	2778	
**#10**	#1 AND #2 AND #4	993	
**#11**	#1 AND #2 AND #5	1300	
**#12**	#1 AND #2 AND #6	888	
**#13**	#1 AND #2 AND #7	549	
**#14**	#1 AND #2 AND #8	751	
**#15**	#9 OR #10 OR #11 OR #12 OR #13 OR #14	5356	
**#16**	#15<br>AND DT=(Article)<br>AND LA=(English)<br>AND DOP=1900-01-01/2026-05-31	4514	
**#17**	AB=(<br>(<br>(SOD1 OR TARDBP OR "TDP-43" OR FUS OR C9orf72)<br>NEAR/5<br>(<br>mutat*<br>OR mutant*<br>OR variant*<br>OR "repeat expansion*"<br>OR "hexanucleotide repeat*"<br>OR "gain-of-function"<br>OR "gain of function"<br>OR "loss-of-function"<br>OR "loss of function"<br>)<br>)<br>OR "GGGGCC repeat*"<br>OR "G4C2 repeat*"<br>OR "C9orf72 repeat expansion*"<br>)	5726	
**#18**	#1 AND #2 AND #17	3026	
**#19**	#9 OR #18	4264	
**#20**	#19 OR #10 OR #11 OR #12 OR #13 OR #14	6273	Union of final mechanism-domain searches
**#21**	#20<br>AND DT=(Article)<br>AND LA=(English)<br>AND DOP=1900-01-01/2026-05-31	5363	Final B set; Article; English; DOP through May 31, 2026

S1F. ScienceDirect: intervention and ALS mechanism searches

Set	Exact search strategy	Results	Notes

Set	Exact search strategy	Results	Notes
**A1**	"amyotrophic lateral sclerosis"<br>AND<br>(<br>"traditional Chinese medicine"<br>OR "Chinese herbal medicine"<br>OR "Chinese patent medicine"<br>OR "herbal medicine"<br>OR "medicinal plant"<br>OR "plant extract"<br>OR phytochem*<br>OR "natural product"<br>)	50	Broad TCM/botanical intervention search
**A2-1**	"amyotrophic lateral sclerosis"<br>AND<br>(<br>"Wen-Pi-Tang"<br>OR Huolingshengji<br>OR "Wen-Shen-Jian-Pi"<br>OR genistein<br>OR oxymatrine<br>OR fisetin<br>OR honokiol<br>OR apigenin<br>)	12	Targeted candidate-intervention query 1
**A2-2**	"amyotrophic lateral sclerosis"<br>AND<br>(<br>oryzanol<br>OR resveratrol<br>OR "tetramethylpyrazine nitrone"<br>OR kaempferol<br>OR celastrol<br>OR lycorine<br>OR baicalein<br>OR gintonin<br>)	42	Targeted candidate-intervention query 2
**B1**	"amyotrophic lateral sclerosis"<br>AND<br>(<br>mutation<br>OR mutant<br>OR "repeat expansion"<br>OR "protein misfolding"<br>OR "protein aggregation"<br>)<br>AND<br>(<br>"in vivo"<br>OR "in vitro"<br>OR model<br>)	915	Research articles; B-search log records 1900-2026
**B2**	"amyotrophic lateral sclerosis"<br>AND<br>(<br>neuroinflammation<br>OR microglia<br>OR astrocyte<br>OR "glial activation"<br>)<br>AND<br>(<br>"in vivo"<br>OR "in vitro"	362	Research articles; B-search log records 1900-2026

Set	Exact search strategy	Results	Notes
	OR model<br>)		
**B3**	"amyotrophic lateral sclerosis"<br>AND<br>(<br>"oxidative stress"<br>OR "reactive oxygen species"<br>OR "mitochondrial dysfunction"<br>OR mitochondrial<br>)<br>AND<br>(<br>"in vivo"<br>OR "in vitro"<br>OR model<br>)	377	Research articles; B-search log records 1900-2026
**B4**	"amyotrophic lateral sclerosis"<br>AND<br>(<br>proteostasis<br>OR autophagy<br>OR "ubiquitin proteasome"<br>OR "endoplasmic reticulum stress"<br>OR "unfolded protein response"<br>)<br>AND<br>(<br>"in vivo"<br>OR "in vitro"<br>OR model<br>)	133	Research articles; B-search log records 1900-2026
**B5**	"amyotrophic lateral sclerosis"<br>AND<br>(<br>excitotoxicity<br>OR glutamate<br>OR EAAT2<br>OR "calcium dysregulation"<br>)<br>AND<br>(<br>"in vivo"<br>OR "in vitro"<br>OR model<br>)	207	Research articles; B-search log records 1900-2026
**B6**	"amyotrophic lateral sclerosis"<br>AND<br>(<br>"neuromuscular junction"<br>OR "axonal transport"<br>OR "axonal dysfunction"<br>OR denervation<br>)<br>AND<br>(<br>"in vivo"<br>OR "in vitro"<br>OR model<br>)	185	Research articles; B-search log records 1900-2026

Notes. PubMed field tags: [Mesh], Medical Subject Headings; [tiab], title/abstract; [lang], language. Web of Science field tags: TS, Topic; TI, Title; AB, Abstract; DT, document type; LA, language; DOP, date of publication. Asterisks (*) indicate truncation. NEAR/n indicates proximity searching in Web of Science. ScienceDirect B searches were executed as six separate mechanism-domain searches because of platform query constraints. The supplied ScienceDirect B search log records the limits as research articles from 1900 to 2026.
